# Assessing the effect of complex ground types on ground‐dwelling arthropod movements with video monitoring: Dealing with concealed movements under a layer of plant residues

**DOI:** 10.1002/ece3.9072

**Published:** 2022-07-11

**Authors:** Blanche Collard, Philippe Tixier, Dominique Carval, Claire Lavigne, Thomas Delattre

**Affiliations:** ^1^ INRAE Avignon France; ^2^ CIRAD, UPR GECO Montpellier France; ^3^ GECO, Univ Montpellier, CIRAD Montpellier France

**Keywords:** insect, mesocosm, monitoring, movement ecology, spatial heterogeneity, video tracking

## Abstract

Understanding the effect of ground types on foraging movements of ground‐dwelling arthropods is a key step to managing their spatial distribution as required for successful conservation biological control. Indeed, fine movements at the centimeter scale can strongly influence the foraging ability of pest predators. However, because radio frequency identification or harmonic tracking techniques are not yet suitable for small species and video tracking focuses on uniform and light backgrounds, foraging movements have rarely been studied in relation to ground types. We present a method to track a ground‐dwelling arthropod (the earwig *Euborellia caraibea*) at night, walking on two contrasted ground types: bare soil and soil partly covered with a stratum of banana plant residues allowing individuals to hide periodically. The tracking of individuals within these ground types was achieved by infrared light, tagging individuals, video treatments, and semi‐automatic cleaning of trajectories. We tested different procedures to obtain segments with identical durations to quantify speeds and sinuosities. These procedures were characterized by the junction time gap between trajectory fragments, the rediscretization time of trajectories, and whether or not to use interpolation to fill in missing points in the trajectories. Earwigs exhibited significantly slower and more sinuous movements on soil with banana plant residues than on bare soil. Long time gaps for trajectory junction, extended rediscretization times, and interpolation were complementary means to integrate concealed movements in the trajectories. The highest slowdown in plant residues was detected when the procedure could account for longer periods under the residues. These results suggest that earwigs spent a significant amount of time concealed by the residues. Additionally, the residues strongly decreased the earwigs' movement. Since the technical solutions presented in this study are inexpensive, easy to set up, and replicate, they represent valuable contributions to the emerging field of video monitoring.

## Graphical abstract

We present a method to monitor and characterize the movement of ground‐dwelling arthropods in mesocosms with realistic ground covers. The method, based on infra‐red video tracking and trajectory processing, made it possible to track night‐time movements that were partially concealed by plant residues. It is generic, simple and highly reusable.

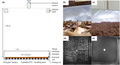

## 1 INTRODUCTION

Ground type quality and spatial configuration are key factors for managing the spatial distribution of organisms for conservation purposes or improving the efficacy of conservation biological control. At the landscape scale, the effect of ground type and spatial distribution on dispersal have been extensively studied (Baguette & Van Dyck, 2007; Haddad et al., 2015), resulting in the development of management practices like movement corridors (Peng et al., 2017; Russell et al., 2018).

In contrast, at the patch scale, foraging movements of ground‐dwelling arthropods (Bell, 1991) have primarily been studied in relation to the distribution of trophic resources (Kareiva & Odell, 1987; Valeix et al., 2009) but not the ground type distribution. Yet, there is evidence that ground type could influence the foraging movement of ground‐dwelling arthropods, by providing a wider and more complex range of resources, such as favorable microclimatic conditions or protection from predators, or by deterring movement by increasing its risks and costs. A typical example is the reluctance of biological control agents to move inside the agricultural plot despite the presence of trophic resources, a frequently proposed explanation for failures in conservation biological control (Albrecht et al., 2020; Al Hassan et al., 2013). Individual‐based models and a meta‐analysis suggest that the interaction between movement behavior and ground type spatial distribution could solve this problem (Albrecht et al., 2020; Collard et al., 2018; Delattre et al., 2019). Therefore, new data on the effect of ground type on patch‐scale foraging movement are necessary for improving conservation biological control at this scale.

Analyzing movements inside an agricultural plot requires collecting high‐resolution data on a small spatial scale, which raises technical obstacles. Mark–Recapture (M‐R) studies may provide insights on inter‐patch movements but generally lack the resolution needed to document intra‐patch movements especially for small ground‐dwelling species (e.g., arthropods) that represent a high proportion of species of agronomic interest. M‐R techniques using radio frequency identification (RFID) active tags are limited to the relatively large species that can handle the chip weight (1 to 0.2 g; Kissling et al., 2014). Moreover, these techniques provide coarse‐grained data (Kissling et al., 2014; Noskov et al., 2021; Vinatier et al., 2010). Harmonic radar seems to hold the best promises for the future, with small tags (6–20 mg; Kissling et al., 2014) and high‐resolution data over a 1 km range. However, this technique has yet to be proven functional in complex vegetation covers (Daout et al., 2017; O'Neal et al., 2004). Its complex development process strongly reduces its immediate availability.

An alternative to in situ remote sensing techniques resides in mesocosm studies, in which one or several ground types (e.g., earthy soil, sand, and plants) can be replicated in an enclosed arena, and the behavior of the target organism is monitored by video (Reynolds & Riley, 2002). Since mesocosm studies allow for high‐resolution data collection, they are well suited for studying foraging behaviors. However, they generally focus on diurnal movements on a uniform and light background to preserve a high contrast with the targets and allow automatic movement tracking. Some more advanced computing methods such as deep learning have been employed to track individuals in more complex or in situ environments and simultaneously track several individuals (Bernardes et al., 2021; Imirzian et al., 2019; Romero‐Ferrero et al., 2018). Nevertheless, to our knowledge, the slightly more heterogeneous and realistic backgrounds were studied in open areas with no vegetation layer, filmed in a rather tight shot allowing only short movements to be recorded (Bjerge et al., 2021; Kindvall et al., 2000) and rarely addressed nocturnal movements (but see Imirzian et al., 2019).

Observing the effect of diverse ground types on movements using heterogeneous mesocosms and video monitoring is challenging, because not all ground types provide good contrast, especially at nighttime, with some concealing parts of the movement paths. Thus, the difficulties of these types of studies reside in (1) separating the target animal from the background by image analysis and (2) reconstructing and analyzing the scattered paths caused by complex three‐dimensional strata (e.g., shelters above ground).

In this study, we developed a method to track ground‐dwelling arthropods in mesocosms that mimic real agricultural ground types including one with two strata that conceals them periodically. This method aimed to investigate how their movement could be affected by the ground type quality, that is, whether it can cause a difference in their sinuosity or their speed. Because the upper stratum could directly affect the movement of these arthropods, even more so if it conceals them, special attention has been given to consider the concealed movements in the analyses. We applied this method to analyze movements of the earwig *Euborellia caraibea* (Hebard), an endemic polyphagous predator of *Cosmopolites sordidus* (Germar) in the Caribbean islands (Brindle, 1971; Carval et al., 2016; Mollot et al., 2014) in mesocosms mimicking two banana field ground types, bare soil, and banana plant residues. Preliminary experiment showed that *E. caraibea* is nocturnal (Appendix A) and seems to prefer the banana plant residues (Collard, pers. data), likely due to the humidity and shelter they provide (Burr, 1939). By using infrared light, different temporal resolutions, and different hypotheses on the behavior of concealed earwigs, we were able to address the problem of nighttime and partially concealed earwig movements.

## 2 MATERIALS AND METHODS

The movement of 17 and 18 *E. caraibea* (including five males per group) was tracked one at a time on one of two ground types: bare soil and banana plant residues, respectively. In this study, the recording of one individual on one ground type represents a replicate.

### 2.1 Capture and laboratory maintenance of *Euborellia caraibea*

*E. caraibea* adults were caught in a field at the Petit Morne site in Martinique (14°37′N, 60°58′W). Species and sex determinations of individuals were performed according to Brindle (1971). Before its movements were tracked, each individual was kept in a 6‐cm‐diameter non‐hermetic box at 25°C (12:12, Light:Dark) in the laboratory for 5 to 34 days. Each box contained a shelter constructed of corrugated cardboard wrapped around a vegetable sponge soaked with distilled water to maintain a constant level of humidity and a water resource. A 1 cm^3^ cube of food (based on Guennelon et al., 1981) was provided once a week.

This device allowed us to keep *E. caraibea* earwigs alive for a relatively long time compared with the duration of the experiments. Out of the 67 earwigs kept in the laboratory until the end of the experiment—35 of which were used for this study—only 12 died.

### 2.2 Experimental mesocosms design

**FIGURE 1:**
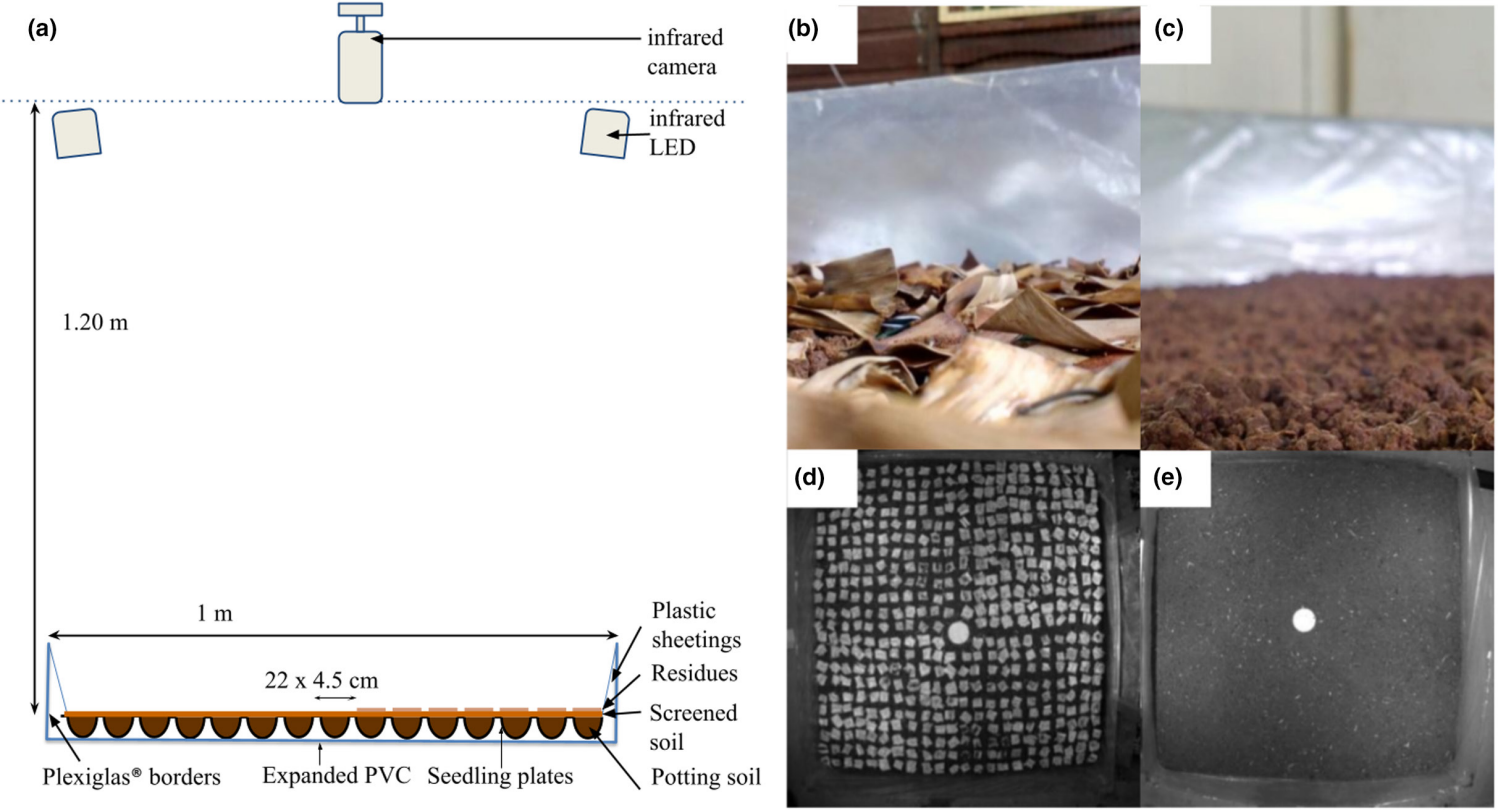
Experimental design for monitoring *Euborellia caraibea* movements in heterogeneous environments: (a) arena diagram, (b, d) photographs of the “residues” arena, (c, e) photographs of the “bare soil” arena. (d, e) Images extracted from infrared nighttime videos

Two 1 × 1 m arenas were built from a square base of 1 m^2^ expanded PVC and 20 cm high Plexiglas edges (Figure 1a) set up at the CIRAD facilities in Martinique (CAEC; 14°37′N, 60°58′W). One arena mimicked a ground with banana plant residues (hereafter referred to as “residues”; Figure 1b,d), and the second arena mimicked a ground of bare soil (hereafter referred to as “bare soil”; Figure 1c,e).

Each arena contained 22 × 22 cells of 4.5 cm side length made from seedling plates, filled with potting soil, and sprinkled with soil from a CIRAD insecticide‐free experimental banana field (“Rivière lézarde” site, 14°39′N, 60°58′W) sifted with a 0.5 cm mesh. In order to prevent the earwigs from escaping, seedling plates were sealed together and connected to the tops of the Plexiglas edges using transparent plastic sheetings coated with talcum powder. For the “residues” arena, 3 × 3 cm units of overlaid dried banana leaves were placed evenly over the bare soil and fixed to a 1 × 1 m wire mesh, with free space between them to allow the detection of the earwig's movement (Figure 1a,d). Only solvent‐free hot glue, wire and tape were used in both arenas. Circular white platforms of 6.5 cm diameter were positioned in the center of each arena to accommodate the earwig in its shelter at the beginning of the experiment.

Each arena was placed under one Trendnet TV‐IP310PI infrared camera (3.2 megapixels, 2048 × 1536), and four projectors containing 48 infrared LED each, were placed on the corners of each arena to provide more uniform lighting (Figure 1a). Red or infrared lights are commonly used to observe the behavior of earwigs (Diehl & Meunier, 2018) or other nocturnal insects, as they are considered invisible to insects (Dias et al., 2012; Drees et al., 2008; Heise, 1992). Infrared lights were preferred, as they are even less likely to have an impact on nocturnal insect behaviors (Allema et al., 2012). The two arenas, video devices and infrared lights were placed in a climatic chamber maintained at 25°C and equipped with white light LEDs (Linear power led, 25 W/4000 K, 2500 LM; Lamentin) providing a 12:12 (L:D) photoperiod (Figure 1a). The light spectrum of similar white light LED can be found in Sharakshane (2017). The two cameras filmed the arenas in parallel using a network switch, a Synology DS216 NAS and its Surveillance Station software.

The cameras created an image distortion depending on their distance from the arenas. It was estimated that 1 cm of the object corresponded to an average of 6.82 pixels over the entire arena with a standard error of ±0.01 cm (see details in Appendix B).

### 2.3 Movement monitoring

Individuals were chosen following their order of capture to homogenize the time spent in captivity. Each day of experiment, two earwigs were randomly assigned to the two arenas.

The shelter containing the chosen individual was placed on the white platform in its arena at 5:00 p.m. on the day of the experiment. The cameras then filmed the arenas from 5:30 p.m. to 8:00 a.m. (i.e., including the entire night period, which started at 6:00 p.m. and ended at 6:00 a.m.). This period corresponds to the estimated activity period for *E. caraibea* under natural outdoor lighting conditions (see the preliminary experiment in Appendix A). The earwigs left the departure platform at various times between 6:06 p.m. and 9:14 p.m., always after the light had been switched off. Since we tracked 17 individuals on bare soil and 18 individuals on residues, we obtained 35 × 14.5 h of videos (resolution 1024 × 768 pixels; 1 frame.s^−1^) between April 26 and May 29, 2017. Only nighttime recordings were used in the rest of this study.

**FIGURE 2:**
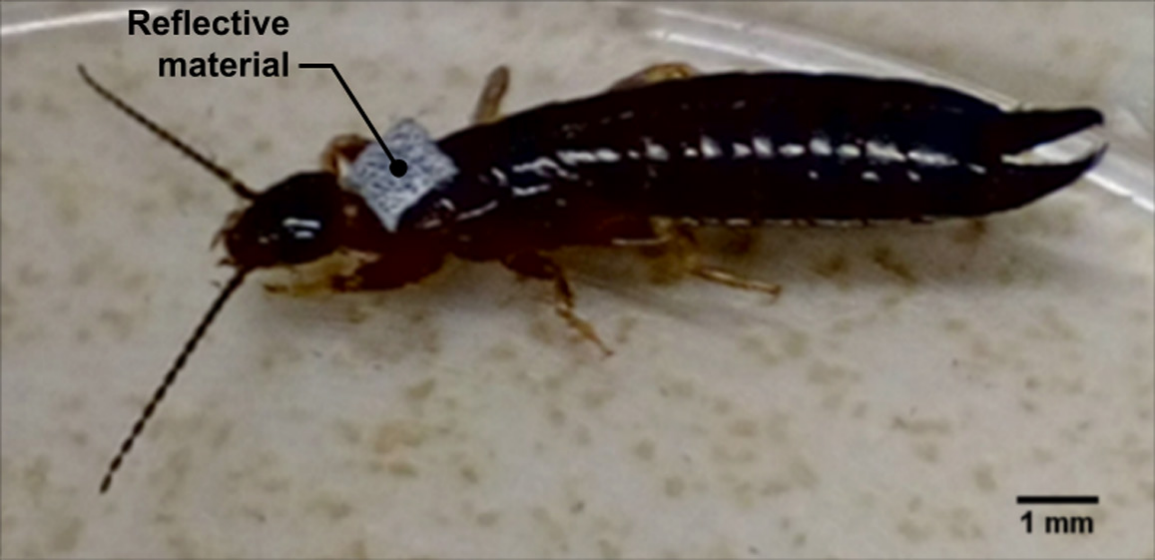
Picture of a tagged earwig *Euborellia caraibe* (personal picture)

Each tested individual was tagged with a 1 × 1 mm square of reflective material (~1 mg, SKU Ref. HEBBR09001; Lecyclo) to allow the infrared light reflection enhancing the visibility of the individuals in the arena. The tag was fixed to the earwig's pronotum with a strong adhesive (cyanoacrylate; Super Glue®) at least 24 h before testing (Figure 2; see detailed tagging protocol in Appendix C). The tagged earwigs showed no visible changes in movements, survival (all tagged earwigs that were recovered after the experiment survived for at least 8 days) and reproduction (some tagged females laid eggs).

The day after the experiment, the arenas were transferred outdoors, the residue grid was removed (for the “residues” treatment), and the bare soil was exposed to the sun to remove or, at a last resort, kill individuals from previous experiments and any organisms that might have entered the arena. A total of 28 earwigs were removed: 14 out of 17 on the bare soil and 14 out of 18 on the residue arenas.

### 2.4 Trajectories extraction from videos

**FIGURE 3:**
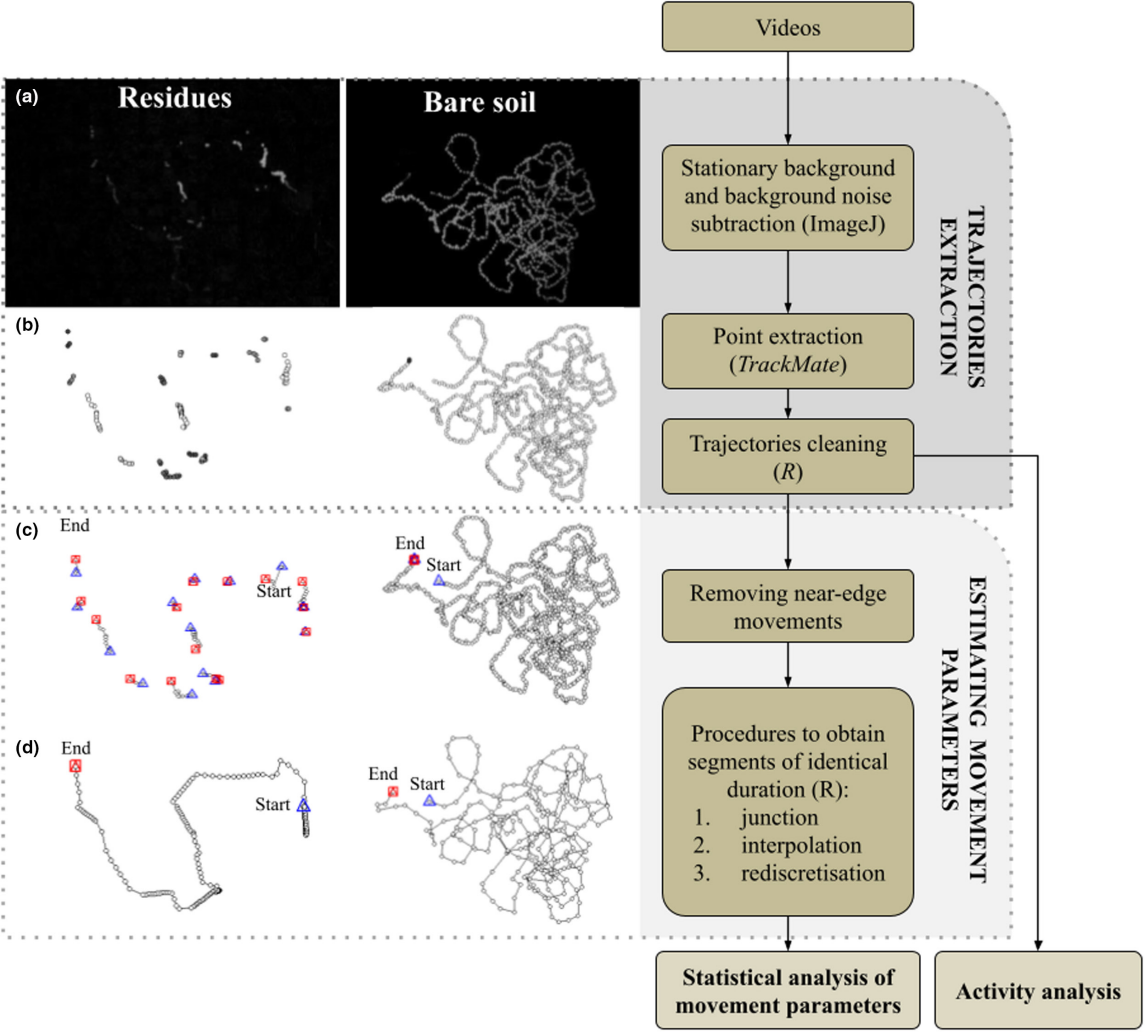
Successive steps to extract trajectories and estimate movement parameters. Each step is illustrated with a 30‐min‐long data selection from both “residues” and “bare soil” treatments. (a) Images representing the maximum intensity obtained for each pixel over a video of 30 min after stationary background and background noise subtraction. (b) Locations extracted via the TrackMate plugin and after cleaning with R software. Examples of trajectories obtained with a specific procedure: (c) a short junction (criteria 10 s, 50 pixels) with a rediscretization at 1 s without interpolation, and d. a long junction between trajectories (criteria 5 min, 50 pixels) and a rediscretization at 5 s with interpolation (for definitions, see Section 2.4)

We developed a method to extract trajectories from videos to characterize the movements (i.e., speed, sinuosity, and earwig activity) in both ground types (Figure 3a,b). The trajectories are defined as a time series of locations of one individual (= points), and each point is characterized by its coordinates and time. Points were grouped into a given trajectory if separated by less than a time gap *t*_g_ and a distance gap *d*_g_ (hereafter referred to as junction criteria) (Tinevez et al., 2017).

The experimental design of this study posed some specific problems that made the extraction of trajectories from the videos difficult, including (i) significant background noise caused by heterogeneous backgrounds and the infrared light and (ii) the simultaneous presence of several individuals in an arena for some replicates where a previous individual had not been found and removed. This methodological problem was a consequence of the presence of soil in the arenas, making it difficult to recover individuals at the end of the experiment. To account for these issues, we used (i) a technique to subtract background and residual noise from the videos and (ii) semi‐automated *cleaning* of the trajectories in R to eliminate conflicting and interacting trajectories.

The videos were treated twice to increase contrast between the moving individuals and the heterogeneous background. The first treatment consisted in subtracting an image of the stationary background from each image of the video. Images summarizing the background were created by calculating the median value (intensity) of each pixel over every 30 min of video. This treatment produced images with the tag of moving individuals in light gray–white on a black background. Those images still contained residual noise in the background that may have been caused by microvariations in light intensity, micromovements in the substrate, or by noise in the camera's infrared light sensors. The second treatment consisted in subtracting an image of this background noise, approximated from the sum of pixel intensities over every 30 min of video. The sum of intensities reached a high value for the pixels that frequently recorded low intensities (dark gray static background noise) and a low value for the pixels that remained black or exhibited rare periods with high intensities (light gray moving individuals). This treatment thus preserved more contrasting black and white images by decreasing pixel intensities mostly for background noise and only slightly for moving individuals (see Figure 3a and videos extracts in Appendix D).

After video treatments, the trajectories were extracted using ImageJ2, an image processing software (Schindelin et al., 2012; version 1.51) and its *TrackMate* plugin (Tinevez et al., 2017; version 3.6.0). *TrackMate* plugin uses a DoG detector algorithm (Difference of Gaussian) and a Simple LAP tracker algorithm (Linear Assignment Problem) (based on Jaqaman et al., 2008). DoG detector allows the detection of small moving points from their size (3 pixels) and a quality threshold (15) (Tinevez et al., 2017). The Simple LAP tracker then associates the points within trajectories (i.e., the junction), one time step after the other, according to the junction criteria *d*_g_ (30 pixels, i.e., ≃ 4.3 cm), and *t*_g_ (5 s) without signal.

**FIGURE 4:**
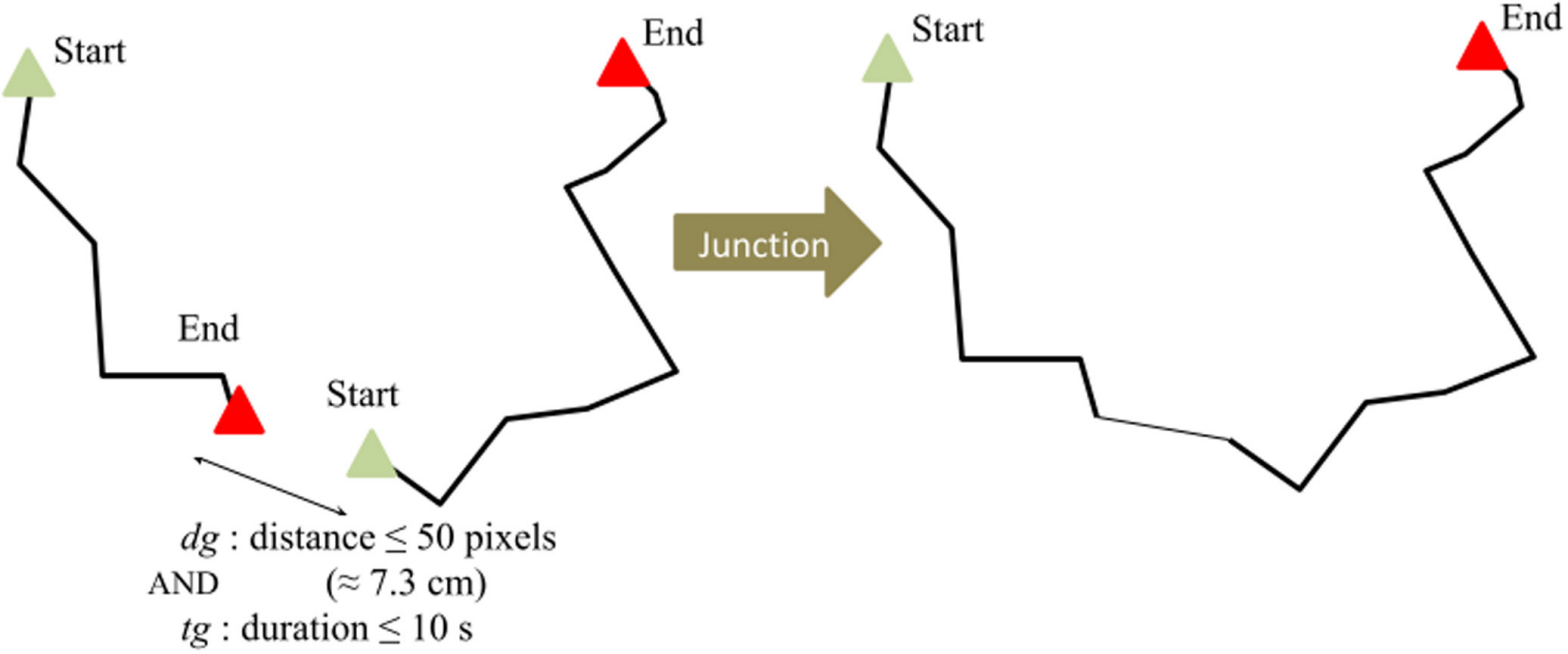
Diagram of the junction of two trajectories according to junction criteria *d*_g_ (distance gap) and *t*_g_ (time gap)

A semi‐automated *cleaning* treatment (see diagram in Appendix E) was performed with the R software (R Core Team, 2018; version 3.6.3) to eliminate conflicting and interacting trajectories. Conflicting trajectories are trajectories that occur simultaneously and that are either false positive (being derived from residual background noise) or real trajectories from other earwigs moving simultaneously. The treatment involved basic automated processes for removing conflicting points under certain circumstances (e.g., when the number of conflict points was small) and visual observations and manual editing of trajectories to remove residual conflicting trajectories otherwise. For example, when trajectories of several earwigs were observed in an arena, the trajectory which was the most consistent with the anterior and posterior trajectories was retained (except in one case where two trajectories, distant from each other by less than 5 cm, were both deleted). Most of conflicting trajectories were removed with automated processes, and only few trajectories had to be cleaned manually per individual (mean ± standard deviation: 2.0 ± 5.4% of trajectories; max of 34 trajectories). This indicated that most of the conflicting trajectories were generated by residual background noise. Once the conflicts were removed and the confusion between individuals and noise was no longer observed, the trajectories were reconstructed with less restrictive junction criteria (*d*_g_ = 50 pixels and *t*_g_ = 10 s) (Figures 3b and 4).

### 2.5 Estimating movement variables

We developed a method to estimate comparable movement variables, that is, the time series of distances and turning angles calculated from segments with identical durations, in both ground types (Figure 3c,d). Two successive points within a trajectory define a segment. A distance is the length of a segment. A turning angle is calculated between the direction of a segment and the direction of the previous segment. Therefore, it takes two successive segments to obtain a turning angle. Due to the disappearance of the tag and missing points within the trajectories in the “residues” arena, segments with an identical duration were rare. The method relies on the design of different procedures to obtain segments of identical duration. These procedures varied in their time resolution and were based on different hypotheses on the behavior of concealed earwigs. Each procedure resulted in a set of movement variables for each individual.

#### 2.5.1 Removing near‐edge movements

To estimate movement variables that were not biased by the arena's edges, we removed all trajectory points within 35 pixels (≈5.1 cm) of the arena borders (Figure 3). This treatment split some trajectories into several trajectories. This operation was always performed after conducting the junction operation for specific time and distance gaps (see next paragraph).

#### 2.5.2 Procedures to obtain segments of identical duration

**FIGURE 5:**
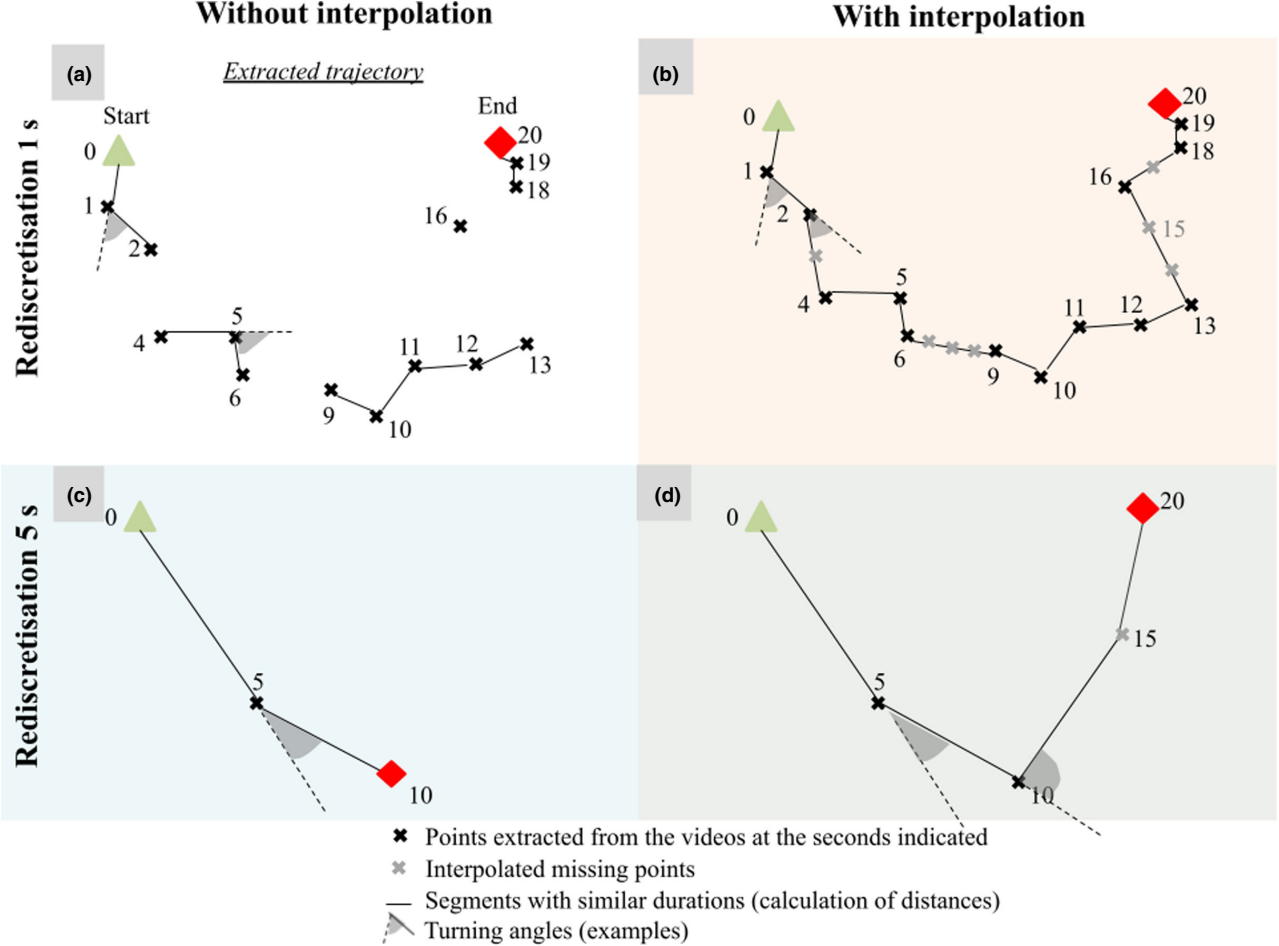
Trajectory procedure scheme for two rediscretization values with or without interpolation: rediscretisation at 1 s without (a) and with (b) interpolation and rediscretisation at 5 s without (c) and with (d) interpolation. The segments for calculating distances and examples of turning angles are shown for each procedure. Interpolation was performed before rediscretization. Consequently, point 15 (d) is taken from the interpolated point 15 (b) and not by linear interpolation between points 10 and 20

A procedure transformed the extracted trajectories into movement variables by means of three different operations: a junction, an interpolation, and a rediscretization of the trajectories (cf. Figures 4 and 5). Each procedure has a corresponding parameter value for each operation (e.g., Figure 3c,d). All operations were performed with the R software (R Core Team, 2018; version 3.6.3) and the *adehabitatLT* package (Calenge, 2006; version 0.3.25).

The junction operation consisted of reconstructing trajectories with a less restrictive junction time gap (*t*_g_) (Figure 4). The interpolation operation involved placing one point for each missing point within trajectories (Figure 5), utilizing linear interpolation between the previous and following extracted points (see interpolation function proposed by *adehabitatLT*). By filling in the missing trajectory points, interpolation allowed the disappearance periods to be included in the speed calculation. The rediscretization operation consisted in modifying the fixed duration of the segments required to estimate the movement variables within each trajectory.

In the absence of interpolation, the segments with identical duration were selected from successive direct observations corresponding to the selected rediscretization time (Figure 5a,c). If there were no observations exactly at the next rediscretisation time, the segment and consequently the distance and the turning angle were not calculated. In the case of interpolation (Figure 5b,d), rediscretization consisted of placing all missing points with interpolation and selecting the segments corresponding to the chosen rediscretization times. Therefore, each dataset of movement variables obtained by a specific procedure was based on a specific way of obtaining segments with identical duration.

#### 2.5.3 Sensitivity of movement variables to operation values

Choices made for the three main operations used to obtain segments of identical duration (junction, interpolation, and rediscretization) may affect the values of movement variables describing the paths (i.e., distances and turning angles). Long junction values (*t*_g_) allow the inclusion into the trajectories of longer times spent under the residues but may lead to a higher occurrence of missing values within the trajectories. Every rediscretization value reveals the individual movement at a different scale and requires a different interpretation. Short rediscretization values indicate the detailed individual trajectory and permit the calculation of mobility parameters like maximum velocity. Long rediscretization values reveal individual net displacement over the corresponding period, allowing the inclusion into the trajectories of longer times spent under the residues in the limits permitted by the dimensions of the arena. Linear interpolation makes it possible to keep all the information in a trajectory but artificially translates time spent under the residues into a straight displacement of equivalent velocity. This can cause underestimation of average speed and sinuosity, for short rediscretization in particular. Because of the strong impact interpolation can have on sinuosity, the calculation of turning angles was only performed on trajectories without interpolation.

To investigate those potential effects, we tested the impact of procedures on movement variables with all combinations of the following values: (i) junctions (*t*_g_) of 10 s, 30 s, 1 min and 5 min; (ii) rediscretizations of 1, 5, 10, 20, 30, 40, 50, and 60 s, (iii) with and without interpolation.

### 2.6 Comparison between ground type

#### 2.6.1 Individual activity

The activity during the night of the experiment was considered only for individuals that were known to have been alone in the arena and which were found at the end with their tag still in place. This represented 9 and 7 individuals for bare soil and residues, respectively. The activity was measured from the entire recorded movements, that is, all “cleaned” trajectories (Figure 3).

We calculated the amount of movements per night per individual (= number of seconds an individual was observed in movement, later termed “apparent intensity of activity”), the number of minutes that an individual was observed with movement (at least 1 s recorded, termed “estimated intensity of activity”) and the time elapsed between the first and last movement of an individual (termed “total activity period”). As residues potentially concealed a significant portion of the movements, the two last measures were performed to limit this information loss bias and better compare activity between the two ground types.

#### 2.6.2 Statistical analysis of movement variables

We tested the effect of residues on travel speed and sinuosity by comparing the movement variables of trajectories (distances and turning angles) between the two ground types. We considered the autocorrelation of successive segments in our analyses by testing the effect of ground types for different rediscretization values (i.e., at different time scales) and taking an individual resolution for our statistical analyses (Fieberg et al., 2010). Individuals released on the same day were considered as independent because they could not interact and external conditions were controlled by the climatic chamber. Individuals that had too little data to have a correct estimate of their movement variables were removed from analyses (see below).

Distances were compared between ground types using datasets of all procedures with at least 10 individuals with at least 10 distance records per ground type. Statistical analyses were performed using mixed linear models (package *lme4*; Bates et al., 2015; version 1.1–26) with individuals as a random effect and a square root transformation of distances expressed in pixels ($\sqrt{\text{distance}}$) to address the overdispersion of the model residuals. Model residuals were inspected for dispersion, uniformity, and outliers with the “DHARMa” R package (Hartig, 2020; version 0.3.3.0). The effect of the individual's sex and its interaction with ground type was removed from the model since they had no significant effect on distance (see analysis in Appendix F).

**FIGURE 6:**
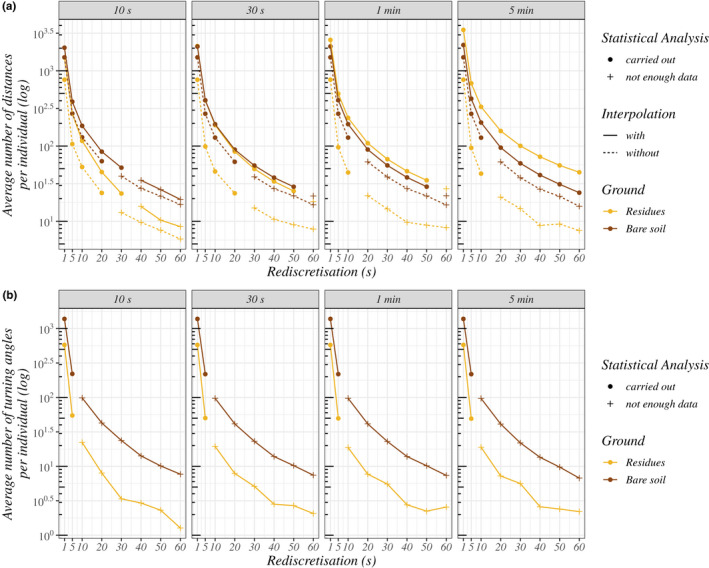
Average number of movement variables sampled per individual according to ground type (color) and procedure. (a, b) The number of distances and turning angles, respectively. The gray banner of the four subsections indicates the junction time gaps. The full circles represent the procedures that were used for the statistical comparison. The plus symbols represent the procedures with too few individuals with enough values of movement variables

Turning angles were compared between ground types for all procedures without interpolation and with a junction of 10 s comprising at least 10 individuals with at least 10 turning angles records per ground type. Only one junction time gap was tested because, in the absence of interpolation, the junction time gap does not affect the turning angles used for analysis (Figure 6b). Individual‐based statistics were used to test the effect of ground type on the concentration of turning angles (1—the variance of turning angles around the mean). Concentration is a measure of the sinuosity of a trajectory, ranging from 0 to 1, with low values corresponding to sinuous trajectories. Statistics developed on R by Pewsey et al. (2013) were used. These analyses rely on the non‐parametric test statistics of Wallraff (1979) and allow the comparison of two circular statistical distributions for the concentration. A randomization test was performed on the test statistics. The statistical distribution under H_0_ was constructed by calculating 1000 statistics obtained after random permutations of the individuals between the ground types to account for the differences in numbers of calculated angles among individuals. The *p*‐values were calculated by comparing the observed statistics with the statistical distribution under the null hypothesis H_0_ of similar values in the two ground types.

## 3 RESULTS

### 3.1 Activity

The apparent intensity of activity appeared to be lower on average on the residues than on the bare soil (inter‐individual mean ± standard deviation, Residues: 1.5 ± 0.8 h, Bare soil: 2.5 ± 2.0 h). We found no such large difference when comparing the estimated intensity of activity (inter‐individual mean ± standard deviation, Residues: 5.9 ± 2.1 h, Bare soil: 5.1 ± 2.7 h) or the total activity period (inter‐individual mean ± standard deviation, Residues: 9.4 ± 2.1 h, Bare soil: 10.0 ± 1.0 h). All measures of activity revealed a highly variable activity pattern (intensity and distribution over time) depending on the earwig's identity and not linked to ground type. The detailed activities of each individual are presented in Appendix G.

### 3.2 Movements variables

#### 3.2.1 Samples generated according to procedures

The trajectories selected for the movement analysis (i.e., excluding near‐edge movements) represented 31.9 ± 18.6% of the total seconds of movement (inter‐individual mean ± standard deviation).

We did not get enough distances to allow statistical comparison of distances for 23 (over 64) procedures (Figure 6a). The average number of segments sampled (= distances) per earwig varied largely depending on the procedure (from 5.6 to 3454.5 segments; Figure 6a). The number of segments was larger for procedures with short rediscretization values, interpolation, and long time gaps for junction (*t*_g_) (starting at *t*_g_ = 30 s). The number of segments was also very variable among the earwigs (from 5 to 1268 segments per earwig for the reference procedure). Segment statistics per individual and per ground type corresponding to data used for statistical analyses for all procedures are given in Appendix H.

Similar effects of procedures on the number of turning angles were found with the difference that interpolation was not used and turning angles were always fewer (Figure 6b). We did not obtain enough data to allow statistical comparison of turning angles for 24 (over 32) procedures, only procedures with rediscretization values of 1 and 5 s analyzed.

#### 3.2.2 Speed

Speeds were calculated from distances as the ratio between distances and the rediscretization value and are presented to simplify the comparison between the different rediscretization values. During their movements, earwigs reached a maximum velocity of 2.19 ± 0.84 cm.s^−1^ (inter‐individual mean ± standard deviation calculated from trajectories with rediscretization at 1 s without interpolation), with a maximal speed of 4.93 cm.s^−1^ registered for an individual on residues.

**FIGURE 7:**
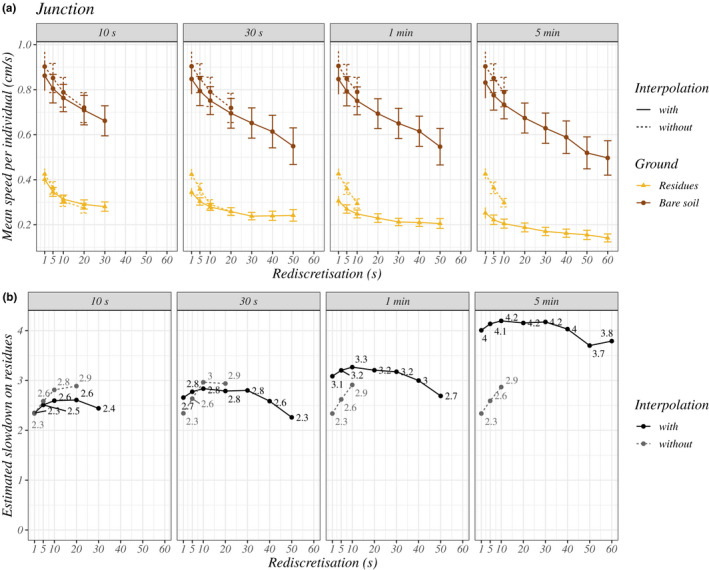
Effect of ground type on the speed of earwigs according to procedures. (a) Mean speeds per earwig (inter‐individual mean ± standard errors) according to ground type (yellow or brown lines), junction (horizontal division), rediscretization (horizontal axis) and interpolation (solid or dotted lines). (b) Estimated ratio of speeds on bare soil versus residues. These ratios were calculated with the predicted distances extracted with the “effects” R package (Fox & Weisberg, 2018; version 4.2–0). All procedures represented here show a significant effect of ground type on speed (all *p*‐values <10^−3^)

All statistical analyses, regardless of the procedure, show a significant effect of ground type on distances (all *p*‐values <10^−3^). The earwigs' mean speeds were estimated 2.3 to 4.2 times slower on residues than on bare soil (Figure 7; see detailed statistical results in Appendix I). For example, the mean speed was 0.36 ± 0.11 cm.s^−1^ (inter‐individual mean ± standard deviation) on residues versus 0.85 ± 0.27 cm.s^−1^ on bare soil, for a procedure with few assumptions, that is, junction of 10 s, without interpolation and rediscretization of 5 s. Estimated slowdowns on residues varied depending on the procedure (Figure 7b). Stronger slowdowns were estimated with long time gaps for junction (starting from 1 min), intermediate rediscretizations (10–30 s), and interpolation. Junction only affected slowdown for procedures with interpolation. In particular, allowing a junction of 5 min with interpolation strongly increased the estimated slowdown. Rediscretization had different effects with and without interpolation. Without interpolation, rediscretizations of 5 s and more increased the estimated slowdown. With interpolation, increasing rediscretization values (starting from 30 s) tended to decrease slowdown. The standard error associated with model estimates also increased for high rediscretization values (see Appendix I).

#### 3.2.3 Turning angle

**FIGURE 8:**
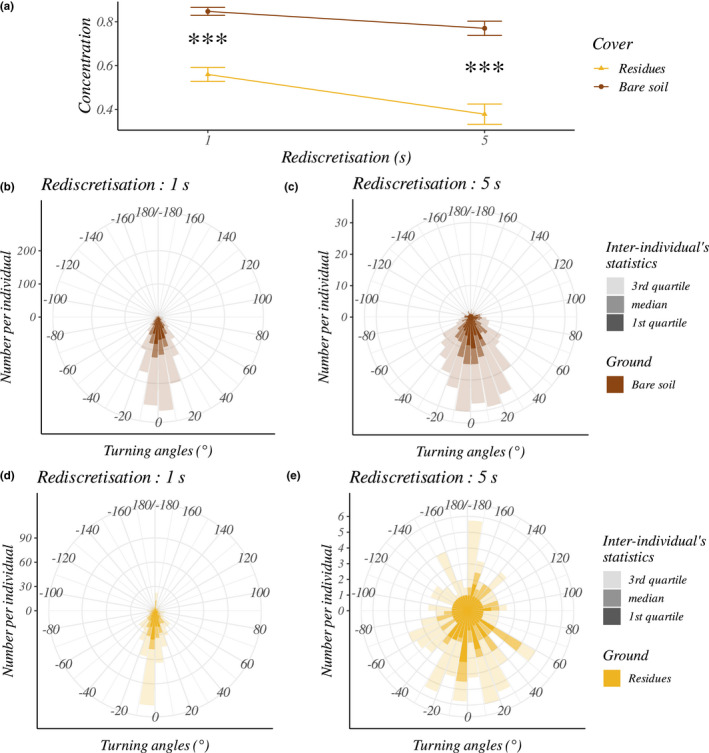
Effect of ground type and rediscretization (1 or 5 s) on the earwig's turning angles distribution (procedures without interpolation and a junction at 10 s). (a) Inter‐individual mean (±standard errors) of turning angles concentration according to ground type (color) and rediscretization value. The significance of the ground type effect on the concentration is represented by: ***p < .001. The circular plots (b–e) indicate inter‐individual median, first quartile, and third quartile of the number of turning angles per earwig for each 10° according to ground type and rediscretization value. Turning angles are expressed in radians which means that positive value are left and negative values are right

The sinuosity of a trajectory increases as the concentration of turning angles decreases. Trajectories obtained for short rediscretization (1 and 5 s) showed significantly higher sinuosities on the residues than the bare soil, caused by smaller concentrations (Figure 8). For the 5 s rediscretization, concentrations were almost twice as large on bare soil (inter‐individual mean ± standard deviation: 0.40 ± 0.16 on residues and 0.77 ± 0.14 on bare soil, *p*‐value <10^−3^, statistic = 219.81; Figure 8a).

## 4 DISCUSSION

Analyzing foraging movement of small organisms at a fine scale, over long periods and on realistic ground is crucial for understanding their spatial and temporal dynamics and help managing them in real agricultural environments. Based on generic video and trajectory processing, we developed a method that made it possible to detect more sinuous and slower movements of the nocturnal earwig *E. caraibea* walking on soil covered by banana leaf residues rather than on bare soil.

### 4.1 Tracking earwigs' movements with an experimental mesocosm and video recording

Our experimental device made it possible to monitor several hours of movement on both ground types. A crucial step of the method was to distinguish earwigs from the background. The use of reflective material on earwigs' pronotum was decisive in increasing the earwigs' contrast. In addition to background and noise subtraction, semi‐automatic cleaning of trajectories was necessary to suppress the remaining noise and to make robust choices between some simultaneous trajectories. The reflective material and video treatments have made the manual editing more manageable given the number of hours, and the movement resolution registered.

The experimental device developed in this study appeared to be more efficient for calculating speeds and highlighting differences in earwig movements for the fine temporal grains (rediscretization ≤30 s). Indeed, we observed a decreasing trend in the estimated slowdown in the residues arena when the values of rediscretization increased (starting at 30 s) with interpolation (Figure 7b). Above 30 s, the dimensions of the arena (<1 m^2^) were probably too small, biasing the sampling of distances toward shorter distances than they might be in open ground. This limitation should be considered when extrapolating the study results to larger spatial and temporal scales, as the distances traveled do not change linearly with sampling time (Morales & Ellner, 2002).

This experimental device opens up the possibility for future analyses on the effects of a wide variety of ground types on the movement of ground‐dwelling arthropods. Additional video treatments or improved tracker algorithms (e.g., Assali et al., 2020) may be required in more complex environments, such as plant cover, where background noise management for trajectory extraction and individual recovery is challenging. In all cases, the spatial distribution of the cover units that could hide individuals should be set to allow the brief but regular appearance of the target species. Previous knowledge of the species and their maximum and mean speed can help optimize the cover design. Similarly to our case, studying cryptic species would probably require improving the contrast between the target and the cover. Thus, choosing an appropriate tag is critical and depends on the species targeted, the ground type tested, and the camera device chosen. In the case of diurnal species, there are more possibilities for distinguishing individuals from the background. Indeed, recording in visible light results in less noise than infrared light (Semenishchev et al., 2018) and standard cameras under visible light return three values per pixel, providing more possibilities to find colors (not just intensities) that distinguish individuals from the background (Sebastian et al., 2010; Wu et al., 2020 preprint). The standard infrared camera used in this experiment returns only one value per pixel (intensity), suggesting that reflective material is probably the best option for nocturnal species.

In recent years, advances in computational methods, such as deep learning, have enabled advances in insect tracking, resulting in significant progress in counting, species recognition, and movement tracking (Bjerge et al., 2021; Høye et al., 2021). However, these methods are still time‐consuming to develop and require a large amount of data, specific to the particular conditions of a given experiment, to train the algorithm. Although in future we can hope for an improvement and greater transferability of the algorithms developed to a wider range of experiments, the use of simpler but faster methods, such as those developed in this study, still seems to us to be of great relevance.

### 4.2 Characterizing movements with trajectories containing missing points

Most trajectories included times where the location of the earwig was not known (missing points). Even on bare soil, missing points were rare but existed (Appendix J). Missing values within trajectories in our experiment could have several explanations: (i) the individual hid under the soil, (ii) the individual movements were not captured by our device or (iii) the individuals were concealed by the residues. Since individuals hiding under the soil (situation i) were rarely observed, and situation (ii) corresponded to a short and random loss of the earwig's tag tracking (e.g., bad inclinations of the tag toward infrared LED), those missing points should not have significantly influenced the quantification of movement on bare soil.

We were interested in the period during which movements were concealed by residues (situation iii) because they could be the source of significant differences in movement variables between the two ground types. Because situation (ii) could also happen on residues, the time with missing points on residues did not exactly match the time individuals spent under residues (situation iii). However, in our experiment, the missing points between residues are unlikely to have affected the estimation of movement variables, particularly slowdown. Indeed, if we had lost track of individuals moving between two or more residues, resulting in an artificially long concealed period, these individuals probably traveled distances larger than the distance gap of 7.3 cm, such that the next data point would be the beginning point of a new trajectory. Herein, we used long time gaps for trajectory junction, long rediscretizations of trajectories, and interpolation to include concealed movements in the trajectories used to quantify movement variables.

The duration of junction time gaps determined the maximum duration of concealed movements included in the trajectories. Long junction time gaps were necessary to account for long concealed movements (up to 5 min). These long junction time gaps affected the quantification of speed and sinuosity only when (i) they were combined with interpolation (because numerous successive interpolated points were added), and (ii) rediscretization was longer than the junction time gaps (because concealed periods shorter than this time gap could be included within a segment).

Varying the rediscretization value without interpolation allowed to include concealed movements while making few assumptions, because only observed movements were taken into account. Short rediscretizations were adapted to analyze speeds and sinuosity on a fine time scale (1–5 s, Figures 7 and 8) because they provided enough data to perform statistical analyses without interpolation (Figure 6a). Some slightly longer rediscretizations (10 and 20 s) generated enough data to compare speeds between ground types including longer concealed movements in trajectories (up to 19 s). However, because the inclusion of these longer concealed movements was only possible for long rediscretization, it was not possible to disentangle the effects of concealed movement from those of time scale on speed and sinuosity. Notably, it has been proposed that each time scale can reflect different behaviors (Nathan et al., 2008; Owen‐Smith et al., 2010). For example, individuals showing (i) a high sinuosity at a time scale but (ii) straight movement at a slightly longer time scale could have indicated a local avoidance of residues (i) while quickly crossing the arena (ii).

Interpolation was another means to include long concealed movements when calculating speeds (and not turning angles). In contrast to long rediscretization, interpolation allowed for the analysis of the concealed movement effects regardless of time scale because it could be combined with both short and long rediscretization values. This approach relies on the ability of the device to successfully track individual movements between residues and proved relevant for the analysis of speeds on larger time scales (5–30 s). The comparison of the speeds estimated with interpolation with speeds from another experiment should be done only under the same data handling procedure because linear interpolation may underestimate real speeds over each ground type.

Missing values are a common problem in animal movement tracking. Even the most advanced techniques such as small remote sensing tags (O'Neal et al., 2004) or deep learning algorithms (Høye et al., 2021) can lose track of the individual when it is hidden by a complex ground cover such as vegetation. By reconstructing trajectories with different temporal resolutions and with different hypotheses on the behavior of concealed earwigs, our method suggests a way to address this problem directly.

### 4.3 Slower and more sinuous movements on the residues

Our study shows that the ground type strongly affected the movement of the earwig *E. caraibea*. Earwigs moved significantly slower on the residues, with speeds estimated to be two to four times slower than on bare soil (Figure 7b). These results are particularly robust because they have been found for all the procedures carrying enough data, whether with interpolated or observed trajectories, long or short junction and long or short rediscretization values.

An increase in the sinuosity of visible movements on residues was also observed for short rediscretizations times of 1–5 s (Figure 8a), that is, the fine movements of the earwig were more sinuous between (or over) the residues than on bare soil. The residues, therefore, seem to have affected the fine movements of the earwigs, speeds, and sinuosity, even when earwigs were visible and not hidden under residues.

Furthermore, a larger slowdown on residues was estimated when concealed movements were included in the speed's calculation (large rediscretization values, Figure 7b or interpolated trajectories with long junctions, Figure 7b), confirming that accounting for concealed movements matters. Earwigs seem to move slower or more sinuously (or both) under the residues than between them. Although procedures with long junction and interpolation must be interpreted with more care, the highest estimated slowdown of the earwigs (up to four times; Figure 7b) obtained for the procedure with interpolation and with the longest junction value (5 min) indicates that earwigs likely stayed hidden for more than 1 min and that this stay strongly slowed down their exploration of the arena. Since the residues hid the earwig's movement from the camera, we expected longer concealed periods on residues. However, the signal loss caused by residues could not account for the strong slowdown by itself (see detailed analysis in Appendix J). Those long periods under residues suggest that residues have not simply slowed down the movements of earwigs as mere obstacles to movement (i.e., a barrier effect) but have caused a behavior change, possibly related to the perception of safety (shelter).

The slowdown and sinuosity of earwigs on residues observed in the present study are consistent with findings on mammals (Beisner & Isbell, 2009; Morales et al., 2004), birds (Da Silveira et al., 2016), and flying insects (Schtickzelle et al., 2007) that show more sinuous and slower movements in their habitats in comparison with unsuitable environments. Such behavior is also supported by the optimal foraging theory according to which animals slow down in food‐rich areas to maximize their yield of food (Andersson, 1978; Bell, 1991) or by the effect of random patterns on animal cognition that increase exploratory behavior (Anselme, 2015). That increased slowdown and sinuosity observed in the absence of food resources suggests that ground type quality per se can have a strong effect on the movement of ground‐dwelling arthropods and confirms the importance to consider the effect of more realistic ground types on their movement. Such knowledge, combined with modeling, can be of great importance to optimize the spatial distributions of their habitats for pest management practices such as conservation biological control (Collard et al., 2018).

## 5 CONCLUSION

To our knowledge, the method described in this study is the first to track the movement of nocturnal ground‐dwelling arthropods in a realistic environment, with a high temporal resolution, an intermediate spatial extent (1 m^2^), and over an entire activity cycle. It allowed us to estimate the slowdown and increase in sinuosity of earwig movements on a complex ground cover. The technical solutions we set up aimed to investigate how a terrestrial arthropod was affected by different ground types. Because they are based on generic principles, we think they are of great interest for the recent growing field of video monitoring and movement ecology. The relatively low cost of the cameras and the free software made it an inexpensive solution, easy to set up, and replicate.

## AUTHOR CONTRIBUTIONS

**Blanche Collard:** Conceptualization (lead); data curation (supporting); formal analysis (lead); investigation (lead); methodology (lead); software (lead); validation (equal); visualization (lead); writing – original draft (lead); writing – review and editing (equal). **Philippe Tixier:** Conceptualization (supporting); data curation (equal); formal analysis (supporting); funding acquisition (equal); investigation (supporting); methodology (supporting); project administration (equal); resources (equal); supervision (equal); validation (equal); writing – review and editing (supporting). **Dominique Carval:** Conceptualization (supporting); formal analysis (supporting); funding acquisition (equal); investigation (supporting); methodology (supporting); project administration (supporting); resources (equal); supervision (supporting); validation (equal); writing – review and editing (supporting). **Claire Lavigne:** Conceptualization (supporting); formal analysis (supporting); funding acquisition (equal); investigation (supporting); methodology (supporting); project administration (equal); resources (equal); software (supporting); supervision (equal); validation (equal); writing – review and editing (equal). **Thomas Delattre:** Conceptualization (supporting); data curation (equal); formal analysis (supporting); funding acquisition (supporting); investigation (supporting); methodology (supporting); project administration (supporting); resources (equal); software (supporting); supervision (equal); validation (equal); visualization (supporting); writing – original draft (supporting); writing – review and editing (equal).

## CONFLICT OF INTEREST

The authors declare no conflicts of interest.

## Supporting information


Appendix S1.

## Data Availability

The data that support the findings of this study are openly available in CIRAD dataverse at https://doi.org/10.18167/DVN1/NPWMUP.

## References

[ece39072-bib-0001] Al Hassan, D. , Georgelin, E. , Delattre, T. , Burel, F. , Plantegenest, M. , Kindlmann, P. , & Butet, A. (2013). Does the presence of grassy strips and landscape grain affect the spatial distribution of aphids and their carabid predators? Agricultural and Forest Entomology, 15, 24–33. 10.1111/j.1461-9563.2012.00587.x

[ece39072-bib-0002] Albrecht, M. , Kleijn, D. , Williams, N. M. , Tschumi, M. , Blaauw, B. R. , Bommarco, R. , Campbell, A. J. , Dainese, M. , Drummond, F. A. , Entling, M. H. , Ganser, D. , Arjen de Groot, G. , Goulson, D. , Grab, H. , Hamilton, H. , Herzog, F. , Isaacs, R. , Jacot, K. , Jeanneret, P. , … Sutter, L. (2020). The effectiveness of flower strips and hedgerows on pest control, pollination services and crop yield: A quantitative synthesis. Ecology Letters, 23, 1488–1498. 10.1111/ele.13576 32808477 PMC7540530

[ece39072-bib-0003] Allema, A. B. , Rossing, W. A. H. , van der Werf, W. , Heusinkveld, B. G. , Bukovinszky, T. , Steingröver, E. , & van Lenteren, J. C. (2012). Effect of light quality on movement of *Pterostichus melanarius* (coleoptera: Carabidae). Journal of Applied Entomology, 136, 793–800. 10.1111/j.1439-0418.2012.01728.x

[ece39072-bib-0004] Andersson, M. (1978). Optimal foraging area—Size and allocation of search effort. Theoretical Population Biology, 13, 397–409. 10.1016/0040-5809(78)90054-0 734622

[ece39072-bib-0005] Anselme, P. (2015). Enhanced exploratory activity in woodlice exposed to random visuo‐tactile patterns. Learning and Motivation, 50, 48–58. 10.1016/j.lmot.2014.09.002

[ece39072-bib-0006] Assali, C. , Bez, N. , & Tremblay, Y. (2020). Raking the ocean surface: New patterns of coordinated motion in seabirds. Journal of Avian Biology, 51, jav.02258. 10.1111/jav.02258

[ece39072-bib-0007] Baguette, M. , & Van Dyck, H. (2007). Landscape connectivity and animal behavior: Functional grain as a key determinant for dispersal. Landscape Ecology, 22, 1117–1129. 10.1007/s10980-007-9108-4

[ece39072-bib-0008] Bates, B. , Mächler, M. , Bolker, B. , & Walker, S. (2015). Fitting linear mixed‐effects models using lme4. Journal of Statistical Software, 67, 48. 10.18637/jss.v067.i01

[ece39072-bib-0009] Beisner, B. A. , & Isbell, L. A. (2009). Movement ecology in a captive environment: The effects of ground substrate on movement paths of captive rhesus macaques, *Macaca mulatta* . Animal Behaviour, 78, 1269–1277. 10.1016/j.anbehav.2009.09.004

[ece39072-bib-0010] Bell, W. J. (1991). Searching behaviour—The behavioural ecology of finding ressources. Chapman and Hall animal behaviour series. Chapman and Hall.

[ece39072-bib-0011] Bernardes, R. C. , Lima, M. A. P. , Guedes, R. N. C. , da Silva, C. B. , & Martins, G. F. (2021). Ethoflow: Computer vision and artificial intelligence‐based software for automatic behavior analysis. Sensors, 21, 3237. 10.3390/s21093237 34067084 PMC8124799

[ece39072-bib-0012] Bjerge, K. , Mann, H. M. R. , & Høye, T. T. (2021). *Real‐time insect tracking and monitoring with computer vision and deep learning*. Remote Sens Ecol Conserv rse2.245. 10.1002/rse2.245

[ece39072-bib-0013] Brindle, A. (1971). The dermaptera of the Caribbean. Studies on the Fauna of Curaçao and Other Caribbean Islands, 38, 1–75.

[ece39072-bib-0014] Burr, M. (1939). Modern work on earwigs. Science Progress (1933‐), 34, 20–30.

[ece39072-bib-0015] Calenge, C. (2006). The package “adehabitat” for the R software: A tool for the analysis of space and habitat use by animals. Ecological Modelling, 197, 516–519. 10.1016/j.ecolmodel.2006.03.017

[ece39072-bib-0016] Carval, D. , Resmond, R. , Achard, R. , & Tixier, P. (2016). Cover cropping reduces the abundance of the banana weevil Cosmopolites sordidus but does not reduce its damage to the banana plants. Biological Control, 99, 14–18. 10.1016/j.biocontrol.2016.04.004

[ece39072-bib-0017] Collard, B. , Tixier, P. , Carval, D. , Lavigne, C. , & Delattre, T. (2018). Spatial organisation of habitats in agricultural plots affects per‐capita predator effect on conservation biological control: An individual based modelling study. Ecological Modelling, 388, 124–135. 10.1016/j.ecolmodel.2018.09.026

[ece39072-bib-0018] Da Silveira, N. S. , Niebuhr, B. B. S. , de Muylaert, R. , Ribeiro, M. C. , & Pizo, M. A. (2016). Effects of land cover on the movement of frugivorous birds in a heterogeneous landscape. PLoS One, 11, e0156688. 10.1371/journal.pone.0156688 27257810 PMC4892584

[ece39072-bib-0019] Daout, F. , Amara, M.‐S. , Schmitt, F. , & Delattre, T. (2017). Mesure de la SER de végétaux en propagation avant—Aspect expérimental, in: Journées Du GDR Ondes 2451 “interférences d'ondes”—Assemblée Générale . https://hal.inrae.fr/hal‐02733581

[ece39072-bib-0020] Delattre, T. , Collard, B. , & Lavigne, C. (2019). Keep your enemies closer: Enhancing biological control through individual movement rules to retain natural enemies inside the field. Web Ecology, 19, 15–26. 10.5194/we-19-15-2019 30925873

[ece39072-bib-0021] Dias, N. , Hassall, M. , & Waite, T. (2012). The influence of microclimate on foraging and sheltering behaviours of terrestrial isopods: Implications for soil carbon dynamics under climate change. Pedobiologia, 55, 137–144. 10.1016/j.pedobi.2011.10.003

[ece39072-bib-0022] Diehl, J. M. C. , & Meunier, J. (2018). Surrounding pathogens shape maternal egg care but not egg production in the European earwig. Behavioral Ecology, 29, 128–136. 10.1093/beheco/arx140

[ece39072-bib-0023] Drees, C. , Matern, A. , & Assmann, T. (2008). Behavioural patterns of nocturnal carabid beetles determined by direct observations under red‐light conditions. In L. Penev , T. Erwin , & T. Assmann (Eds.), Back to the roots and back to the future: Towards a new synthesis amongst taxonomic, ecological and biogeographical approaches in carabidology, proceedings, Pensoft series Faunistica (pp. 409–423). Pensoft Publishers.

[ece39072-bib-0024] Fieberg, J. , Matthiopoulos, J. , Hebblewhite, M. , Boyce, M. S. , & Frair, J. L. (2010). Correlation and studies of habitat selection: Problem, red herring or opportunity? Philosophical Transactions of the Royal Society B: Biological Sciences, 365, 2233–2244. 10.1098/rstb.2010.0079 PMC2894958 20566500

[ece39072-bib-0057] Fox, J. , & Weisberg, S. (2018). Visualizing fit and lack of fit in complex regression models with predictor effect plots and partial residuals. Journal of Statistical Software, 87(9). 10.18637/jss.v087.i09

[ece39072-bib-0025] Guennelon, G. , Audemard, H. , Fremond, J.‐C. , el Idrissi, A. , & Ammari, M. (1981). Progrès réalisés dans l'élevage permanent du Carpocapse (*Laspeyresia pomonella* L.) sur milieu artificiel. Agronomie, 1, 59–64.

[ece39072-bib-0026] Haddad, N. M. , Brudvig, L. A. , Clobert, J. , Davies, K. F. , Gonzalez, A. , Holt, R. D. , Lovejoy, T. E. , Sexton, J. O. , Austin, M. P. , Collins, C. D. , Cook, W. M. , Damschen, E. I. , Ewers, R. M. , Foster, B. L. , Jenkins, C. N. , King, A. J. , Laurance, W. F. , Levey, D. J. , Margules, C. R. , … Townshend, J. R. (2015). Habitat fragmentation and its lasting impact on Earth's ecosystems. Science Advances, 1, e1500052. 10.1126/sciadv.1500052 26601154 PMC4643828

[ece39072-bib-0027] Hartig, F. (2020). DHARMa: Residual diagnostics for hierarchical (multi‐level/mixed) regression models. R package . https://CRAN.R‐project.org/package=DHARMa

[ece39072-bib-0028] Heise, B. A. (1992). Sensitivity of mayfly nymphs to red light: Implications for behavioural ecology. Freshwater Biology, 28, 331–336. 10.1111/j.1365-2427.1992.tb00591.x

[ece39072-bib-0029] Høye, T. T. , Ärje, J. , Bjerge, K. , Hansen, O. L. P. , Iosifidis, A. , Leese, F. , Mann, H. M. R. , Meissner, K. , Melvad, C. , & Raitoharju, J. (2021). Deep learning and computer vision will transform entomology. Proceedings of the National Academy of Sciences of the United States of America, 118, e2002545117. 10.1073/pnas.2002545117 33431561 PMC7812775

[ece39072-bib-0030] Imirzian, N. , Zhang, Y. , Kurze, C. , Loreto, R. G. , Chen, D. Z. , & Hughes, D. P. (2019). Automated tracking and analysis of ant trajectories shows variation in forager exploration. Scientific Reports, 9, 13246. 10.1038/s41598-019-49655-3 31519955 PMC6744467

[ece39072-bib-0031] Jaqaman, K. , Loerke, D. , Mettlen, M. , Kuwata, H. , Grinstein, S. , Schmid, S. L. , & Danuser, G. (2008). Robust single‐particle tracking in live‐cell time‐lapse sequences. Nature Methods, 5, 695–702. 10.1038/nmeth.1237 18641657 PMC2747604

[ece39072-bib-0032] Kareiva, P. , & Odell, G. (1987). Swarms of predators exhibit “Preytaxis” if individual predators use area‐restricted search. The American Naturalist, 130, 233–270.

[ece39072-bib-0033] Kindvall, O. , Nordlander, G. , & Nordenhem, H. (2000). Movement behaviour of the pine weevil Hylobius abietis in relation to soil type: An arena experiment. Entomologia Experimentalis et Applicata, 95, 53–61. 10.1046/j.1570-7458.2000.00641.x

[ece39072-bib-0034] Kissling, W. D. , Pattemore, D. E. , & Hagen, M. (2014). Challenges and prospects in the telemetry of insects. Biological Reviews, 89, 511–530. 10.1111/brv.12065 24106908

[ece39072-bib-0035] Mollot, G. , Duyck, P. F. , Lefeuvre, P. , Lescourret, F. , Martin, J. F. , Piry, S. , Canard, E. , & Tixier, P. (2014). Cover cropping alters the diet of arthropods in a banana plantation: A metabarcoding approach. PLoS One, 9, e93740. 10.1371/journal.pone.0093740 24695585 PMC3973587

[ece39072-bib-0058] Morales, J. M. , & Ellner, S. P. (2002). Scaling up animal movements in heterogeneous landscapes: The importance of behavior. Ecology, 83(8), 2240–2247. 10.1890/0012-­9658(2002)083[2240:suamih]2.0.co;2

[ece39072-bib-0036] Morales, J. M. , Haydon, D. T. , Frair, J. , Holsinger, K. E. , & Fryxell, J. M. (2004). Extracting more out of relocation data: Building movement models as mixtures of random walks. Ecology, 85, 2436–2445. 10.1890/03-0269

[ece39072-bib-0037] Nathan, R. , Getz, W. M. , Revilla, E. , Holyoak, M. , Kadmon, R. , Saltz, D. , & Smouse, P. E. (2008). A movement ecology paradigm for unifying organismal movement research. Proceedings of the National Academy of Sciences of the United States of America, 105, 19052–19059. 10.1073/pnas.0800375105 19060196 PMC2614714

[ece39072-bib-0038] Noskov, A. , Bendix, J. , & Friess, N. (2021). A review of insect monitoring approaches with special reference to radar techniques. Sensors, 21, 1474. 10.3390/s21041474 33672508 PMC7923785

[ece39072-bib-0039] O'Neal, M. E. , Landis, D. A. , Rothwell, E. , Kempel, L. , & Reinhard, D. (2004). Tracking insects with harmonic radar: A case study. American Entomologist, 50, 212–218. 10.1093/ae/50.4.212

[ece39072-bib-0040] Owen‐Smith, N. , Fryxell, J. M. , & Merrill, E. H. (2010). Foraging theory upscaled: The behavioural ecology of herbivore movement. Philosophical Transactions of the Royal Society B: Biological Sciences, 365, 2267–2278. 10.1098/rstb.2010.0095 PMC2894968 20566503

[ece39072-bib-0041] Peng, J. , Zhao, H. , & Liu, Y. (2017). Urban ecological corridors construction: A review. Acta Ecologica Sinica, 37, 23–30. 10.1016/j.chnaes.2016.12.002

[ece39072-bib-0042] Pewsey, A. , Neuhäuser, M. , & Ruxton, G. D. (2013). Circular statistics in R. Oxford University Press.

[ece39072-bib-0043] R Core Team . (2018). R: A language and environment for statistical computing. R Foundation for Statistical Computing.

[ece39072-bib-0044] Reynolds, D. R. , & Riley, J. R. (2002). Remote‐sensing, telemetric and computer‐based technologies for investigating insect movement: A survey of existing and potential techniques. Computers and Electronics in Agriculture, 35, 271–307. 10.1016/S0168-1699(02)00023-6

[ece39072-bib-0045] Romero‐Ferrero, F. , Bergomi, M. G. , Hinz, R. , Heras, F. J. H. , & de Polavieja, G. G. (2018). Idtracker.Ai: Tracking all individuals in large collectives of unmarked animals. Nature Methods, 16, 179–182. 10.1038/s41592-018-0295-5 30643215

[ece39072-bib-0046] Russell, K. N. , Russell, G. J. , Kaplan, K. L. , Mian, S. , & Kornbluth, S. (2018). Increasing the conservation value of powerline corridors for wild bees through vegetation management: An experimental approach. Biodiversity and Conservation, 27, 2541–2565. 10.1007/s10531-018-1552-8

[ece39072-bib-0047] Schindelin, J. , Arganda‐Carreras, I. , Frise, E. , Kaynig, V. , Longair, M. , Pietzsch, T. , Preibisch, S. , Rueden, C. , Saalfeld, S. , Schmid, B. , Tinevez, J.‐Y. , White, D. J. , Hartenstein, V. , Eliceiri, K. , Tomancak, P. , & Cardona, A. (2012). Fiji: An open‐source platform for biological‐image analysis. Nature Methods, 9, 676–682. 10.1038/nmeth.2019 22743772 PMC3855844

[ece39072-bib-0048] Schtickzelle, N. , Joiris, A. , Van Dyck, H. , & Baguette, M. (2007). Quantitative analysis of changes in movement behaviour within and outside habitat in a specialist butterfly. BMC Evolutionary Biology, 7, 4. 10.1186/1471-2148-7-4 17241457 PMC1784076

[ece39072-bib-0049] Sebastian, P. , Vooi Voon, Y. , & Comley, R. (2010). Colour space effect on tracking in video surveillance. International Journal on Electrical Engineering and Informatics, 2, 298–312. 10.15676/ijeei.2010.2.4.5

[ece39072-bib-0050] Semenishchev, E. A. , Voronin, V. V. , & Balabaeva, O. S. (2018). Stitching of IR images into a single content based on data which analysis obtained by an optical camera. In D. A. Huckridge , H. Bursing , & D. L. Hickman (Eds.), Electro‐optical and infrared systems: Technology and applications xv, proceedings of SPIE. Presented at the conference on electro‐optical and infrared systems—Technology and applications XV. Spie‐Int Soc Optical Engineering. 10.1117/12.2326839

[ece39072-bib-0051] Sharakshane, A. (2017). Whole high‐quality light environment for humans and plants. Life Sciences in Space Research, 15, 18–22. 10.1016/j.lssr.2017.07.001 29198310

[ece39072-bib-0052] Tinevez, J. Y. , Perry, N. , Schindelin, J. , Hoopes, G. M. , Reynolds, G. D. , Laplantine, E. , Bednarek, S. Y. , Shorte, S. L. , & Eliceiri, K. W. (2017). TrackMate: An open and extensible platform for single‐particle tracking. Methods, 115, 80–90. 27713081 10.1016/j.ymeth.2016.09.016

[ece39072-bib-0053] Valeix, M. , Loveridge, A. J. , Davidson, Z. , Madzikanda, H. , Fritz, H. , & Macdonald, D. W. (2009). How key habitat features influence large terrestrial carnivore movements: Waterholes and African lions in a semi‐arid savanna of North‐Western Zimbabwe. Landscape Ecology, 25, 337–351. 10.1007/s10980-009-9425-x

[ece39072-bib-0054] Vinatier, F. , Chailleux, A. , Duyck, P.‐F. , Salmon, F. , Lescourret, F. , & Tixier, P. (2010). Radiotelemetry unravels movements of a walking insect species in heterogeneous environments. Animal Behaviour, 80, 221–229. 10.1016/j.anbehav.2010.04.022

[ece39072-bib-0055] Wallraff, H. G. (1979). Goal‐oriented and compass‐oriented movements of displaced homing pigeons after confinement in differentially shielded aviaries. Behavioral Ecology and Sociobiology, 5, 201–225. 10.1007/bf00293306

[ece39072-bib-0056] Wu, M. , Cao, X. , & Guo, S. (2020). Accurate detection and tracking of ants in indoor and outdoor environments. Animal Behavior and Cognition. 10.1101/2020.11.30.403816

