# Supplementary material for: Assessing the effect of complex ground types on ground‐dwelling arthropod movements with video monitoring: Dealing with concealed movements under a layer of plant residues
Source: Ecol Evol. 2022 Jul 11;12(7):10.1002/ece3.9072. doi: 10.1002/ece3.9072 (PMC9271991; doi:10.1002/ece3.9072)
Supplement: Supplementary file 1 — Appendix S1. [file ECE3-12--s001.zip › ECE3_9072_SupplementaryMaterials_2022.06.09.pdf]

Appendices

A. Preliminary experiment - activity range of E. caraibea

Purpose

The purpose of this preliminary experiment was to define the period of activity of *E. caraibea*. It was expected to be nocturnal like many dermaptera (Burr, 1939; Joachim and Weisser, 2015).

Materials and methods

Ten *E. caraibea* were caught (7 females and 3 males) on a plot of old banana plant cultivars at the CIRAD Petit Morne site. These individuals were kept in captivity for 48 hours before the experiment began in a 20 x 30 cm box with wet corrugated cardboard shelters, a water source, plenty of food, a temperature of 25°C and a photoperiod of 18:6.

At the beginning of the experiment on February 20, 2017 at 12:00 pm, the shelters containing all the individuals were placed in the centre of an arena. Earwigs were hidden in the shelters at this time of day and did not leave until nightfall. The arena consisted of a white square base of 1 m² expanded PVC. The 20 cm high Plexiglas edges were coated with talcum powder on the inside of the arena. The device was set outdoors, in a rainproof environment Activity was filmed by a Trendnet TV-IP310PI (3MP) infrared (IR) sensitive camera and 48 IR LED projectors for 4 days continuously. No food was provided during the 4 days in order to observe a foraging activity, only a source of water (moistened sand cup) and the humidity of the shelters were maintained (vegetable sponge). At the end of the experiment, only 9 earwigs were recovered, meaning that one of them either escaped or was cannibalised.

The videos were viewed for 1 minute every 30 minutes over the 4 x 24 hours to monitor the number of earwigs outside the shelters.

Result and conclusion

The *E. caraibea* dermaptera showed nocturnal activity, between 6:00 pm and 8:30 am, starting just after sunset, over periods of 9 to 14 hours (**Fig. B.1**). There was a slight increase in activity on the 2nd day and a

slight decrease in activity from the 3rd day. We also observed that before 6:00 am the majority of the individuals had entered the shelter and that all the individuals had returned when the sun was projecting its rays directly into the arena. It is therefore still possible that *E. caraibea* may have a daily activity if it is in a sufficiently shaded place.

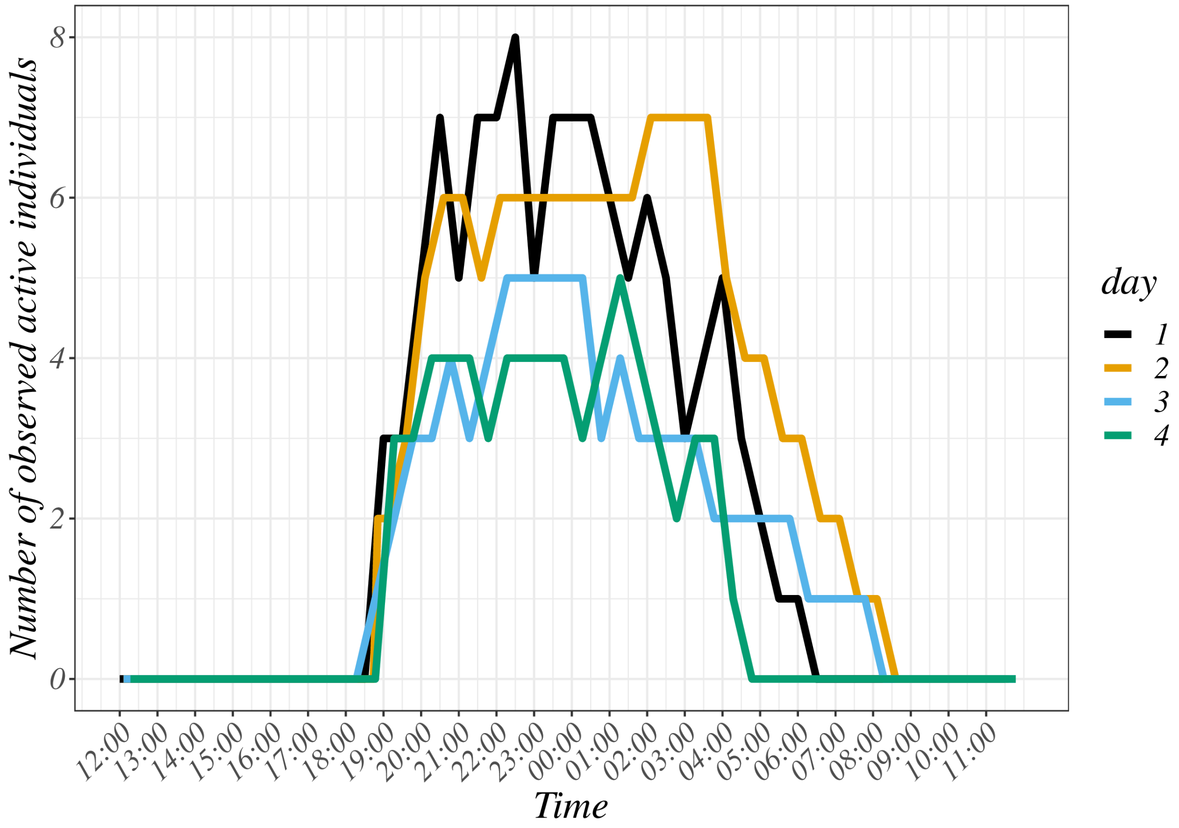

Fig. A.1 : Number of distinct active individuals observed over 1 min of observation every 30 min

B. Image distortion estimation

The cameras used in this experiment created an image distortion depending on their distance from the arenas. This distortion was estimated using a 6.55 cm object placed, photographed and measured (in pixels) in the arena at several distances from the centre and on the edges. Only a slight dilatation of the image in the centre was observed : 1 cm of the object was corresponding to an average of 6.82 ± 0.51 pixels over the entire arena (min: 5.98, max: 7.51). This average was then used to convert the pixels to centimetres (error ± 0.01 cm).

C. Earwigs tagging protocol

Each individual was tagged with a square of reflective material of about 1 x 1 mm (SKU Ref. HEBBR09001, Lecyclo, France) to allow the reflection of the infrared light and enhance the visibility of the individuals in the arena. This tag was fixed at least 24 hours before an individual was tested with a strong adhesive (cyanoacrylate, Super Glue®) on its pronotum (**Fig. 2**). According to knowledge on the earwig *F. auricularia* (Lamb and Wellington, 1974), the application of cyanocrylate on its pronotum avoids its possible negative effects on the individual. In the end, only four of the 28 individuals recaptured after monitoring in the arena had lost their tag.

Materials

- Strong liquid glue (cyanoacrylate, super glue®)
- tag: pieces of reflective material of about 1 x 1 mm
- Petri dish of 5 cm diameter
- 1 cup
- Small piece of wiping paper
- 1 or 2 needles
- Fine tweezers
- (Binocular loupe)

Protocol

1. Prepare a cup with a few drops of strong glue.
2. Put the earwig in a Petri dish in the freezer for 1 min and 30 s to immobilise it while minimising side effects (Lamb and Wellington, 1974)
    - In the meantime, prick the edge of a piece of reflective tissue with the needle in order to manipulate it.
3. Before the earwig moves:
    - Wipe the pronotum with the piece of wiping paper and the fine tweezers
    - Take a drop of Super glue from the cup using a needle

- Place the drop on the pronotum (**Fig. C.1A**)
- Place the tag on the drop using a needle and possibly the tweezers (**Fig. C.1B**)
- Press very slightly with the tweezers (**Fig. C.1C**)

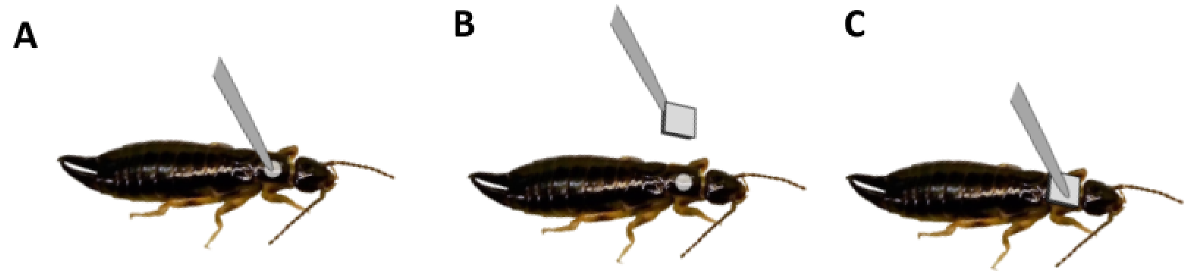

Fig. C.1 : Explanatory diagram for tagging E. caraibea earwigs (personal pictures)

4. As soon as the earwig starts to wake up and bend backwards
    - It is too late to place the tag: if it is not in place, you have to start again from step 2.
    - Flip it to one side and then to the other (**Fig. C.2A**)
    - And place another drop of super glue on each left and right edge, between the reflective tissue and the top of the pronotum (**Fig. C.2B**)

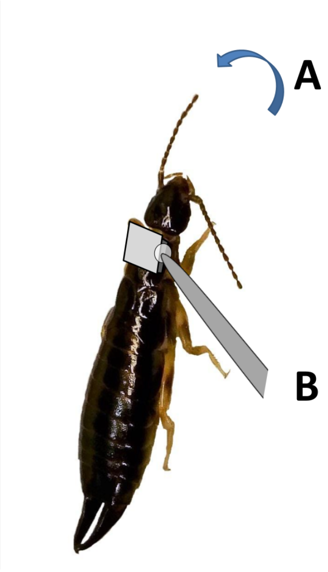

Fig. C.2 : Explanatory diagram for tagging E. caraibea earwigs (personal photo)

- Do not make the super glue below the pronotum overflow on its legs.

An earwig *was excluded from the experiment because the super glue had overflowed on the legs and seemed to interfere with its movement. This precaution is also present in the literature for F. auricularia. Lamb and*

Wellington (1974) recommend that deposition of substances with solvents should be made only on the pronotum or elytra, thus avoiding the potential lethal or paralysing effects of these substances.

- Possibility to use a binocular loupe

This last step (additional side drops) ensured a better hold of the tagging on the earwig.

5. Close the Petri dish
6. Arrange a lid over the Petri dish to protect the earwig from light.
7. Leave the earwig to "rest" for at least 1 hour.
8. Put the earwig back in its box with a clean shelter, in a cardboard compartment that is in good condition so that the tag does not hit the edge and that it is easy to see whether the tagging has held or not the next day.
9. Put food in abundance
10. Leave the earwig box in the breeding room at least 24 hours before the start of the experiment.

D. Videos clips of earwig's movement on both ground types

Two videos are given for each ground type (**Video D.1** and **Video D.2**). For each video, the recording of the arena before and after video treatments (subtraction of background and noise) are given side by side (left : original, right : after treatments). To make the movement more visible, the videos were accelerated. One second of video now corresponds to one minute of the original video.

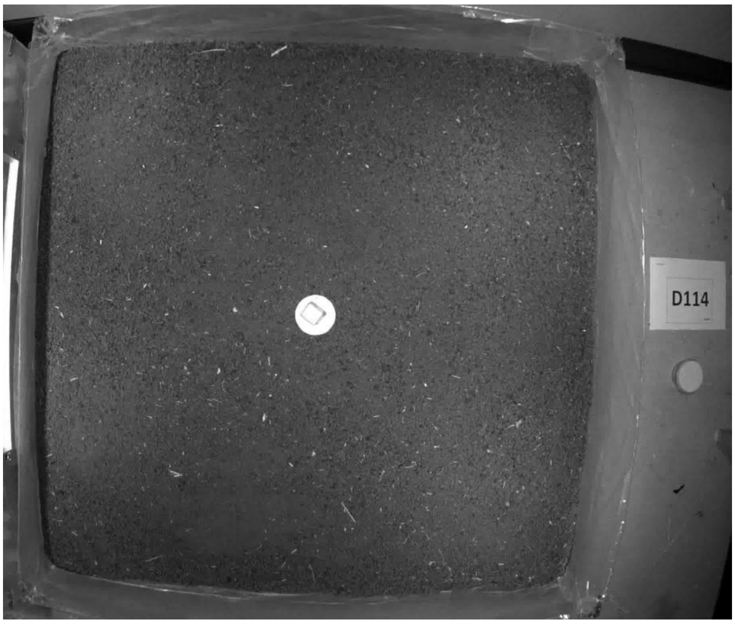

Video D.1 : Video clip on « bare soil » arena for the earwig D114

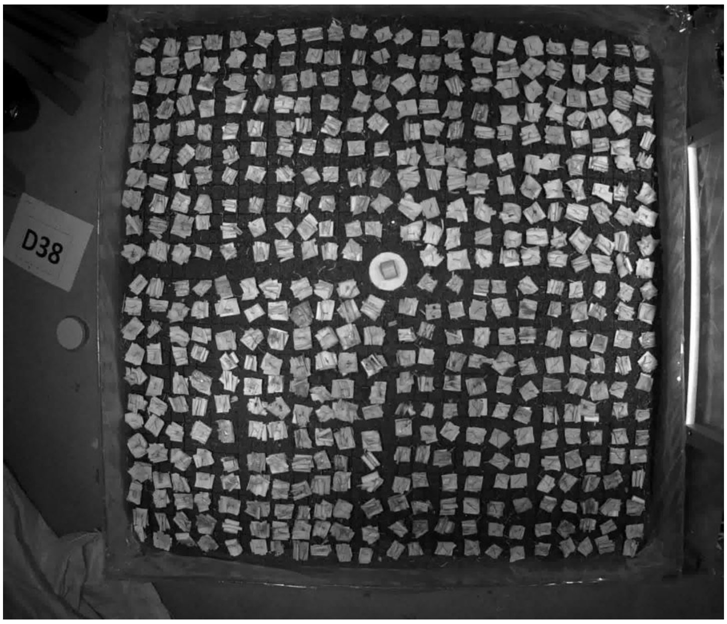

Video D.2 Video clip on « residues » arena for the earwig D38

E. Trajectory cleaning details (with R)

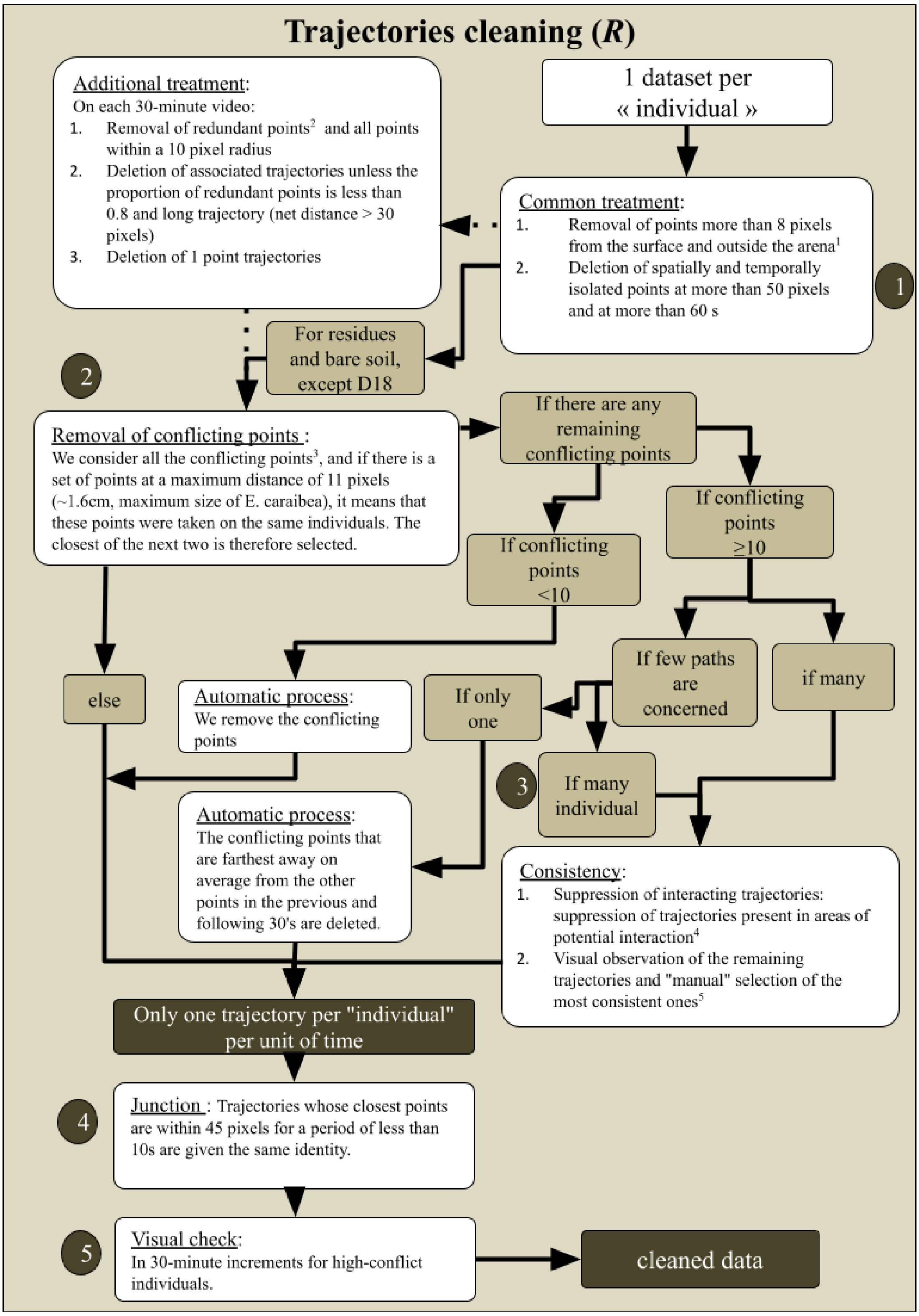

Fig E.1 : Semi-automatic cleaning of trajectories.

1: For each arena and each day of measurement, we created an image (1024 x 768) mapping 4 different areas (outside the arena, borders, surface, white platform) using layers on the GIMP software (GIMP team, 1997; version 2.10.18).

2: redundant points = pixels with more than 20 observed points (high intensities) during 30-minute videos.

3: conflicting points = points are in conflict if they are observed at the same time on different locations

4: interaction area = rectangle containing conflicting trajectories within 35 pixels of each other (~5cm))

5: criteria used = 1) trajectory with the highest ligth intensity of the points, 2) spatial and temporal proximity with previous and following trajectories

6: remaining simultaneous trajectories = two trajectories that happen in parallel (A trajectory starts before the previous ends) without conflicting points. Most of the simultaneous trajectories were managed when finding conflicting points but some of them remained by chance because their points alternated one after the other and never occurred at the same time.

7: two simultaneous trajectories are joined in a unique trajectory if all points taken together meet the time and distance gap criteria (dg = 50 pixels and tg = 10 s)

8: All 2 listed operations were done by order one at a time (“1” and then “2”) and only if the previous operation did not suppress all simultaneous trajectoires.

9: Only one trajectory required “manual” editing.

F. Effect of sex and its interaction with ground type on distance

We tested the effect of the ground type, the sex of the individual and their interaction on distance for the procedure with few assumptions (junction: 10 s, without interpolation, rediscretisation: 5 s). The rediscretisation value of 5 s was chosen visually over a series of trajectories to avoid oversampling (Turchin, 1998). Statistical analyses were performed using mixed linear models (package lme4, version 1.1-26) with individuals as a random effect and a square root transformation of distances expressed in pixels to address their overdispersion.

The speed of earwigs was significantly decreased on residues compared to bare soil (**Table F.1**), with a mean speed of earwigs of 0.36 ± 0.11 cm/s vs 0.85 ± 0.27 cm/s on residues vs bare soil, respectively (inter-individual mean ± standard deviation). No effects of sex or sex*ground type interaction were found. A large part of the distance variance was not explained by the model (1 - conditional $R^2 = 0.54$), but a substantial part was explained by ground type (marginal $R^2 = 0.28$).

Table F.1: Mixed generalized linear model of the effect of ground type, sex and their interaction on distance (pixels) for the reference procedure (rediscretisation : 5 s, junction : 10 s, without interpolation).

Modèles	Estimate	chi²	p-value	Marginal R²	Conditional R²	AIC
distance ~ ground + sex + ground : sex				0.287	0.461	21390.3
ground (ref: residues)	2.16a	61.17	***			
sex	0.19	0.59	0.442			
ground : sex	-0.75	1.73	0.188			
distance ~ ground				0.276	0.462	21388.5
ground (ref: residues)	1.95a	56.61	***			

«***» for a p-value < 0.001

G. Activity of individuals depending on time, ground type and sex

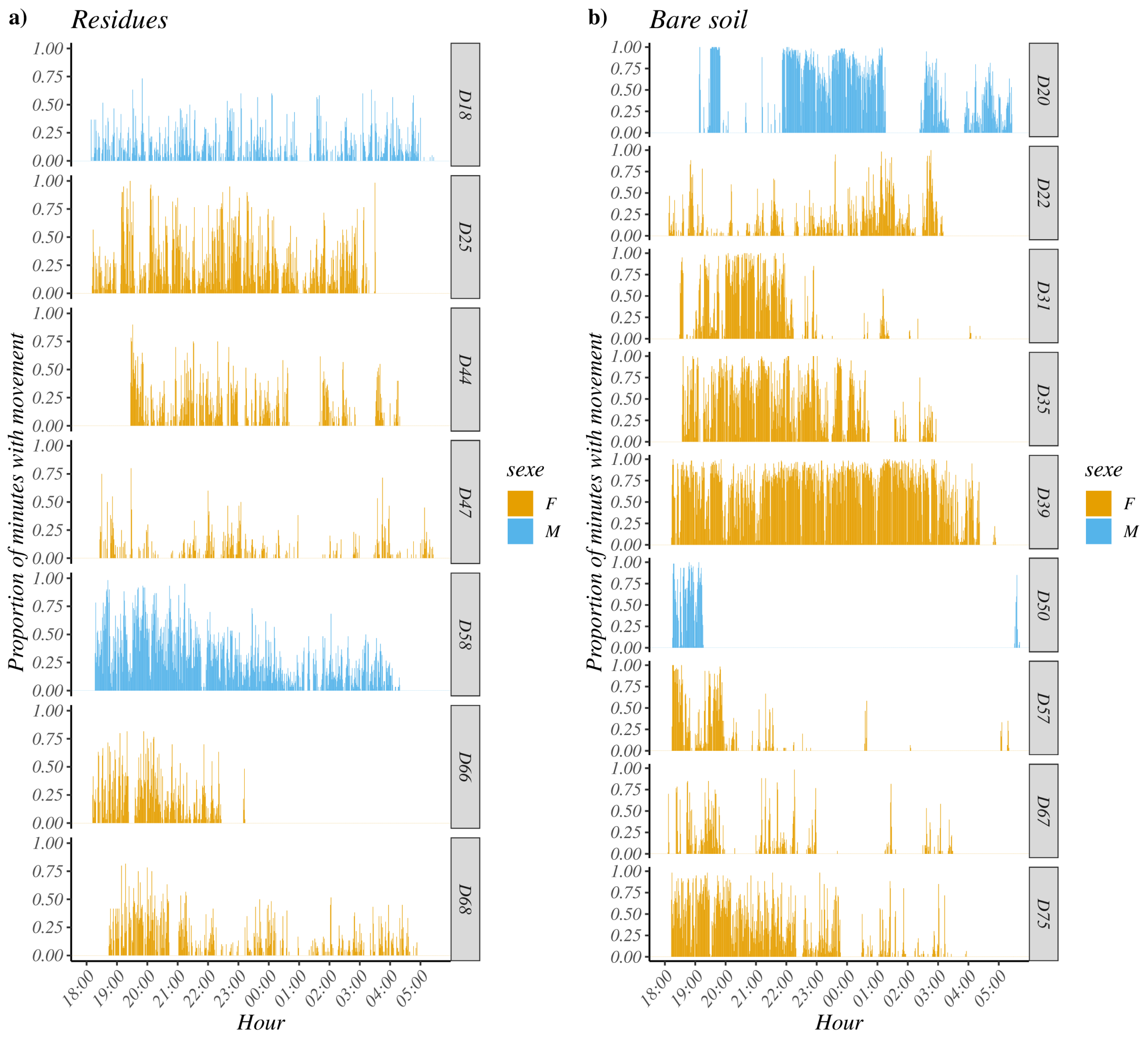

Fig. G.1 : For each individual and minute the coloured bars represent the proportion of seconds with movement. Orange and blue colours represent females and males, respectively.

H. Number of individuals and distances per individual selected for the comparisons of distances between ground types

Table H.1 : Number of individuals and distances per individual selected for the comparisons of distances between ground types

Ground	Junction	Interpolation	Rediscretisation	Number of individuals	Average number of segments per individual	Sd	Min	Max
Residues	10 s	with	1 s	18	1504.83	1991.85	109	9017
Bare soil	10 s	with	1 s	17	2026.88	254973	108	10302
Residues	10 s	without	1 s	18	761.06	1044.63	45	4671
Bare soil	10 s	without	1 s	17	1509.53	1896.25	92	7229
Residues	10 s	with	10 s	17	123.65	177.27	12	785
Bare soil	10 s	with	10 s	17	185.59	242.75	10	959
Residues	10 s	without	10 s	15	62.33	78.41	14	332
Bare soil	10 s	without	10 s	16	138.25	161.08	12	607
Residues	10 s	with	20 s	16	50.44	79.11	10	339
Bare soil	10 s	with	20 s	15	94.67	115.87	13	443
Residues	10 s	without	20 s	12	44347	40.94	11	158
Bare soil	10 s	without	20 s	15	70.6	79.47	12	299
Residues	10 s	with	30 s	12	33.17	52.9	10	199
Bare soil	10 s	with	30 s	14	61.64	73.97	13	276
Residues	10 s	with	5 s	18	268.67	375.22	18	1696
Bare soil	10 s	with	5 s	17	388.76	502.23	21	1986
Residues	10 s	without	5 s	16	118.81	161.54	19	692
Bare soil	10 s	without	5 s	17	270.88	336.43	18	1264
Residues	30 s	with	1 s	18	2129.94	2542.45	168	11383
Bare soil	30 s	with	1 s	17	2101.82	2725.59	108	10946
Residues	30 s	without	1 s	18	761.06	1044.63	45	4671
Bare soil	30 s	without	1 s	17	1509.53	1896.25	92	7229
Residues	30 s	with	10 s	18	190	239.8	16	1073
Bare soil	30 s	with	10 s	17	194.76	259.38	10	1043

Ground	Junction	Interpolation	Rediscretisation	Number of individuals	Average number of segments per individual	Sd	Min	Max
Residues	30 s	without	10 s	15	54.27	74.22	12	312
Bare soil	30 s	without	10 s	16	137	158.98	12	595
Residues	30 s	with	20 s	17	89.12	115.76	15	508
Bare soil	30 s	with	20 s	15	100.8	125.35	13	488
Residues	30 s	without	20 s	11	32.91	41.03	10	152
Bare soil	30 s	without	20 s	15	69.27	77.66	11	290
Residues	30 s	with	30 s	16	55.06	71.91	10	310
Bare soil	30 s	with	30 s	14	65.86	81.26	13	309
Residues	30 s	with	40 s	13	44.54	55.77	10	222
Bare soil	30 s	with	40 s	13	48.38	57.92	12	216
Residues	30 s	with	5 s	18	403.72	496.09	33	2221
Bare soil	30 s	with	5 s	17	405.71	532.47	21	2138
Residues	30 s	without	5 s	16	110.38	153.6	19	655
Bare soil	30 s	without	5 s	17	268.82	332.35	18	1243
Residues	30 s	with	50 s	11	35.82	44.58	11	167
Bare soil	30 s	with	50 s	10	44.8	49.5	11	167
Residues	1 min	with	1 s	18	245962	2873.97	228	12461
Bare soil	1 min	with	1 s	17	2108.35	2740.63	108	11020
Residues	1 min	without	1 s	18	761.06	1044.63	45	4671
Bare soil	1 min	without	1 s	17	1509.53	1896.25	92	7229
Residues	1 min	with	10 s	18	237.22	273.9	21	1186
Bare soil	1 min	with	10 s	17	195.29	260.65	10	1049
Residues	1 min	without	10 s	15	52.73	75.65	12	316
Bare soil	1 min	without	10 s	16	137.06	159.17	12	596
Residues	1 min	with	20 s	17	115.12	132.77	20	566
Bare soil	1 min	with	20 s	15	101.07	126.17	13	492
Residues	1 min	with	30 s	17	70.41	82.97	13	353
Bare soil	1 min	with	30 s	14	66.14	81.95	13	312

Ground	Junction	Interpolation	Rediscretisation	Number of individuals	Average number of segments per individual	Sd	Min	Max
Residues	1 min	with	40 s	16	51.38	60.41	10	252
Bare soil	1 min	with	40 s	14	45.86	57.04	10	218
Residues	1 min	with	5 s	18	496.22	563.04	41	2439
Bare soil	1 min	with	5 s	17	407.12	535.91	21	2155
Residues	1 min	without	5 s	16	107.69	151.78	18	646
Bare soil	1 min	without	5 s	17	268.88	332.53	18	1244
Residues	1 min	with	50 s	14	43.21	48.27	11	194
Bare soil	1 min	with	50 s	10	44.9	49.46	11	167
Residues	5 min	with	1 s	18	3498.39	3713.39	234	15266
Bare soil	5 min	with	1 s	17	2202.82	371939	108	11754
Residues	5 min	without	1 s	18	761.06	1044.63	45	4671
Bare soil	5 min	without	1 s	17	1509.53	1896.25	92	7229
Residues	5 min	with	10 s	18	332.28	361.73	22	1480
Bare soil	5 min	with	10 s	17	205.29	279.26	10	1126
Residues	5 min	without	10 s	15	50.87	73.77	12	308
Bare soil	5 min	without	10 s	16	136.75	158.73	12	594
Residues	5 min	with	20 s	18	158.56	176.48	10	718
Bare soil	5 min	with	20 s	15	107.13	136.16	13	531
Residues	5 min	with	30 s	17	106.06	113.97	17	457
Bare soil	5 min	with	30 s	14	70.57	89.11	13	339
Residues	5 min	with	40 s	17	76.18	83.69	11	334
Bare soil	5 min	with	40 s	13	52.46	64.43	12	240
Residues	5 min	with	5 s	18	682.28	734.72	44	3013
Bare soil	5 min	with	5 s	17	426.35	571.78	21	2303
Residues	5 min	without	5 s	16	105.62	149	18	634
Bare soil	5 min	without	5 s	17	268.24	331.09	18	1243
Residues	5 min	with	50 s	15	65.07	66.29	15	258
Bare soil	5 min	with	50 s	11	45	53.26	10	185
Residues	5 min	with	60 s	15	52.8	54.83	11	210

Ground	Junction	Interpolation	Rediscretisation	Number of individuals	Average number of segments per individual	Sd	Min	Max
Bare soil	5 min	with	60 s	10	37.9	43.34	10	148
Residues	5 min	with	50 s	16	103.12	119.13	10	495
Bare soil	5 min	with	50 s	14	105.5	124.87	14	380
Residues	5 min	with	60 s	15	89.6	100.79	14	411
Bare soil	5 min	with	60 s	14	86.86	103.28	11	314

I. Detailed statistical results for models $\sqrt{distance}$ ~ Ground type

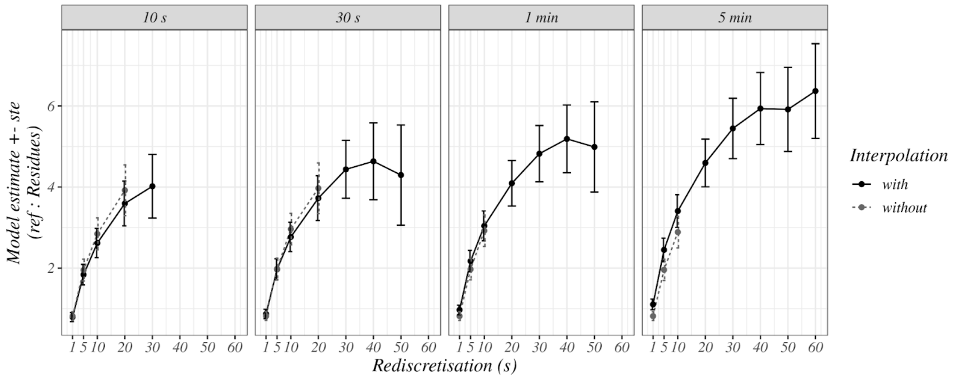

Fig. I.1: Ground type estimates ± standard errors in the models analysing the effect of ground type on the square root transformed distance (distance) for each procedure.

Table I.1 : Statistical analysis values for all models $\sqrt{distance}$ ~ Ground type

Interpolation	Junction	Rediscretisation (s)	Ground type estimate ± ste (ref: Residues)	Intercept estimate ± ste	Chi²	P-value
with	10 s	1	0.793 ± 0.113	1.491 ± 0.079	49.02	2.54 E-12
with	10 s	5	1.84 ± 0.254	3.148 ± 0.177	52.36	4.62 E-13
with	10 s	10	2.62 ± 0.362	4.287 ± 0.256	52.42	4.49 E-13
with	10 s	20	3.595 ± 0.553	5.845 ± 0.387	42.32	7.76 E-11
with	10 s	30	4.019 ± 0.787	7.144 ± 0.581	26.11	3.22 E-07
with	30 s	1	0.872 ± 0.117	1.383 ± 0.081	55.65	8.67 E-14
with	30 s	5	1.971 ± 0.26	2.961 ± 0.181	57.61	3.20 E-14
with	30 s	10	2.77 ± 0.362	4.047 ± 0.252	58.51	2.02 E-14
with	30 s	20	3.727 ± 0.552	5.562 ± 0.378	45.63	1.43 E-11
with	30 s	30	4.436 ± 0.714	6.584 ± 0.488	38.61	5.17 E-10
with	30 s	40	4.635 ± 0.948	7.622 ± 0.67	23.91	1.01 E-06
with	30 s	50	4.296 ± 1.234	8.532 ± 0.853	12.12	4.99 E-04
with	1 min	1	0.97 ± 0.119	1.282 ± 0.083	66.36	3.76 E-16
with	1 min	5	2.175 ± 0.265	2.753 ± 0.184	67.50	2.11 E-16
with	1 min	10	3.043 ± 0.369	3.767 ± 0.256	68.15	1.52 E-16
with	1 min	20	4.094 ± 0.561	5.177 ± 0.384	53.16	3.07 E-13
with	1 min	30	4.823 ± 0.695	6.167 ± 0.467	48.15	3.96 E-12
with	1 min	40	5.188 ± 0.836	7.088 ± 0.57	38.53	5.39 E-10
with	1 min	50	4.989 ± 1.111	7.792 ± 0.717	20.15	7.15 E-06
with	5 min	1	1.106 ± 0.132	1.104 ± 0.092	69.86	6.36 E-17
with	5 min	5	2.45 ± 0.29	2.37 ± 0.202	71.15	3.31 E-17
with	5 min	10	3.408 ± 0.405	3.25 ± 0.281	70.99	3.60 E-17

Interpolation	Junction	Rediscretisation (s)	Ground type estimate ± ste (ref: Residues)	Intercept estimate ± ste	Chi²	P-value
with	5 min	20	4.595 ± 0.588	4.426 ± 0.396	60.97	5.79 E-15
with	5 min	30	5.446 ± 0.744	5.22 ± 0.498	53.63	2.42 E-13
with	5 min	40	5.937 ± 0.89	5.891 ± 0.584	44.50	2.54 E-11
with	5 min	50	5.914 ± 1.037	6.402 ± 0.671	32.52	1.18 E-08
with	5 min	60	6.368 ± 1.169	6.724 ± 0.736	29.68	5.09 E-08
without	10 s	1	0.817 ± 0.107	1.544 ± 0.075	58.08	2.52 E-14
without	10 s	5	1.953 ± 0.268	3.214 ± 0.193	53.26	2.92 E-13
without	10 s	10	2.846 ± 0.394	4.204 ± 0.285	52.10	5.28 E-13
without	10 s	20	3.921 ± 0.627	5.605 ± 0.47	39.17	3.89 E-10
without	30 s	1	0.819 ± 0.106	1.543 ± 0.074	59.35	1.32 E-14
without	30 s	5	1.984 ± 0.263	3.181 ± 0.19	57.04	4.27 E-14
without	30 s	10	2.963 ± 0.387	4.103 ± 0.281	58.51	2.02 E-14
without	30 s	20	3.971 ± 0.625	5.559 ± 0.478	40.38	2.10 E-10
without	1 min	1	0.818 ± 0.107	1.547 ± 0.074	58.87	1.68 E-14
without	1 min	5	1.972 ± 0.261	3.187 ± 0.188	57.09	4.17 E-14
without	1 min	10	2.924 ± 0.387	4.139 ± 0.281	56.97	4.42 E-14
without	5 min	1	0.817 ± 0.107	1.546 ± 0.074	58.89	1.67 E-14
without	5 min	5	1.958 ± 0.262	3.21 ± 0.189	55.78	8.11 E-14
without	5 min	10	2.889 ± 0.39	4.165 ± 0.283	54.90	1.27 E-13

J. Concealed duration depending on ground type and time gap for junction

In this appendix, we investigate the time earwigs spent under residues, thereafter referred to as concealed duration, to see how it affects the speed's calculation. The closest approximation to concealed duration is the periods without movement (missing values) within trajectories. Missing values within trajectories in our experiment can have several explanations : (i) the individual hid in the soil (rarely observed), (ii) the individual movements were not captured by our device (frequent but not long) or (iii) the individuals were concealed by residues (moving or not). As we can consider that situation (i) and (ii) should be similar between bare soil and residues, the comparison of periods without observed movement between the two ground types should help approaching the time earwig spent under residues. Periods without movements were calculated for individuals within part of trajectories that were far enough from the edge and not on the departure platform (see **section 2.4**).

As the residues formed a layer that covered a great portion of the area, it was expected that earwigs disappeared more frequently in the "residues" arena than on bare soil. In order to clarify the actual extra time earwigs may have spent under residues, we looked at the periods without observed movement for individuals on bare soil when concealing artificially parts of trajectories by a false layer of residues, later called "concealed bare soil". The layer of residues was drawn with Gimp software from a picture of an arena with residues and its position towards the bare soil arena in the videos was adjusted for each individual (**Fig. J.1**). Then in R, points of trajectories located on this layer were suppressed to mimic the concealed effect of residues.

For each individual, we calculated the mean and the total time without observed movement within trajectoires (concealed duration). The mean concealed duration provides an estimation of the consecutive concealed duration, which may, on the residues arena, account for the mean time earwigs spend under one residue. The total concealed duration may stand on the residues arena for the total time earwigs spend under residues during one day.

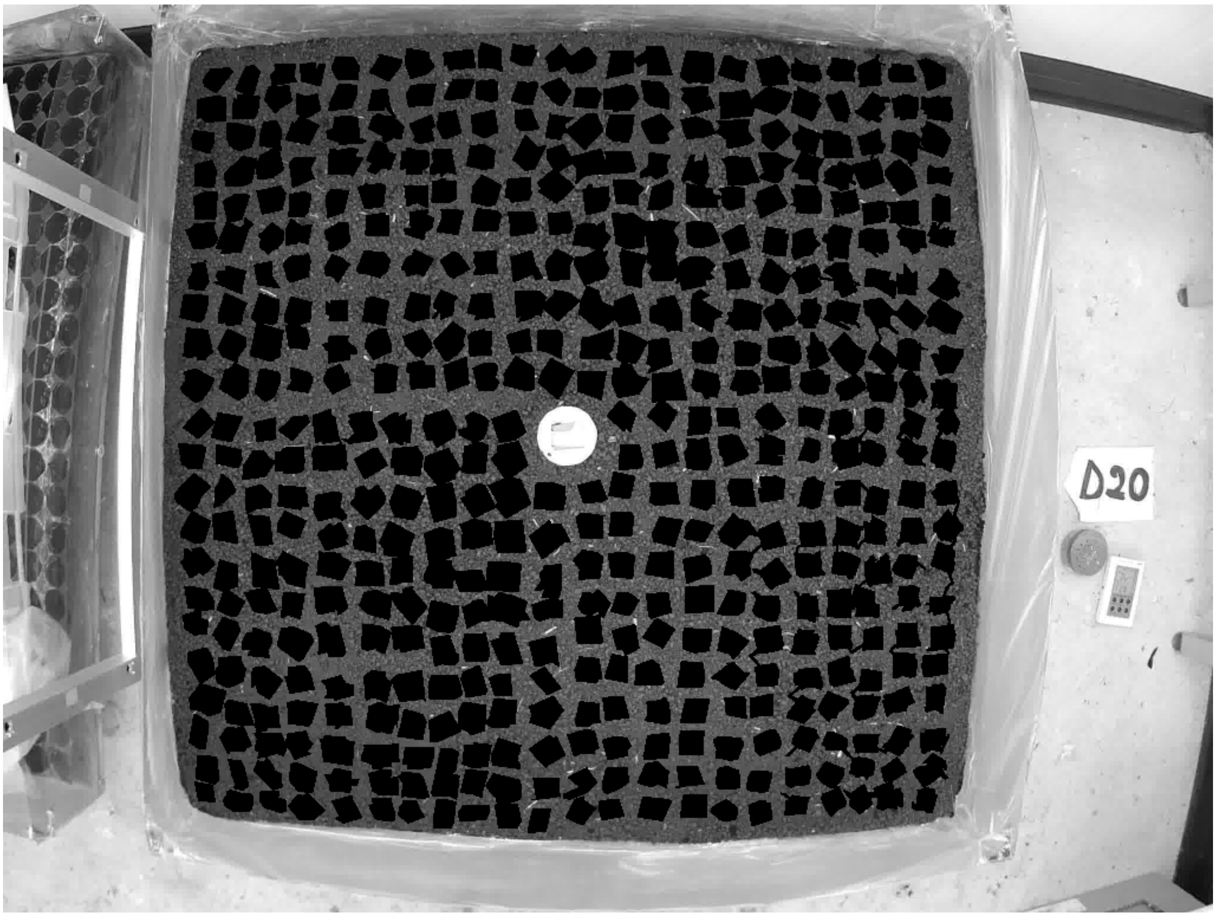

Fig. J.1 : Example of an artificial layer of residues adjusted on a bare soil arena and its departure platform. Points of bare soil trajectories located under the black area would be suppressed for the "Concealed bare soil" modality.

As expected, we found globally a higher concealed duration (mean and total time without observed movement within trajectories) per individual on residues or on concealed bare soil, than on bare soil (**Fig. J.2a** and **J.2b**). Concealed durations were also generally higher on residues than on concealed bare soil. Mean concealed durations per individual were globally higher, even for the shortest junction (10 s ; **Fig. J.2a**). Total concealed durations per individual are globally higher only when junctions of 1 min or more are allowed (**Fig. J.2b**).

Time without movement within trajectories (estimated concealed duration)

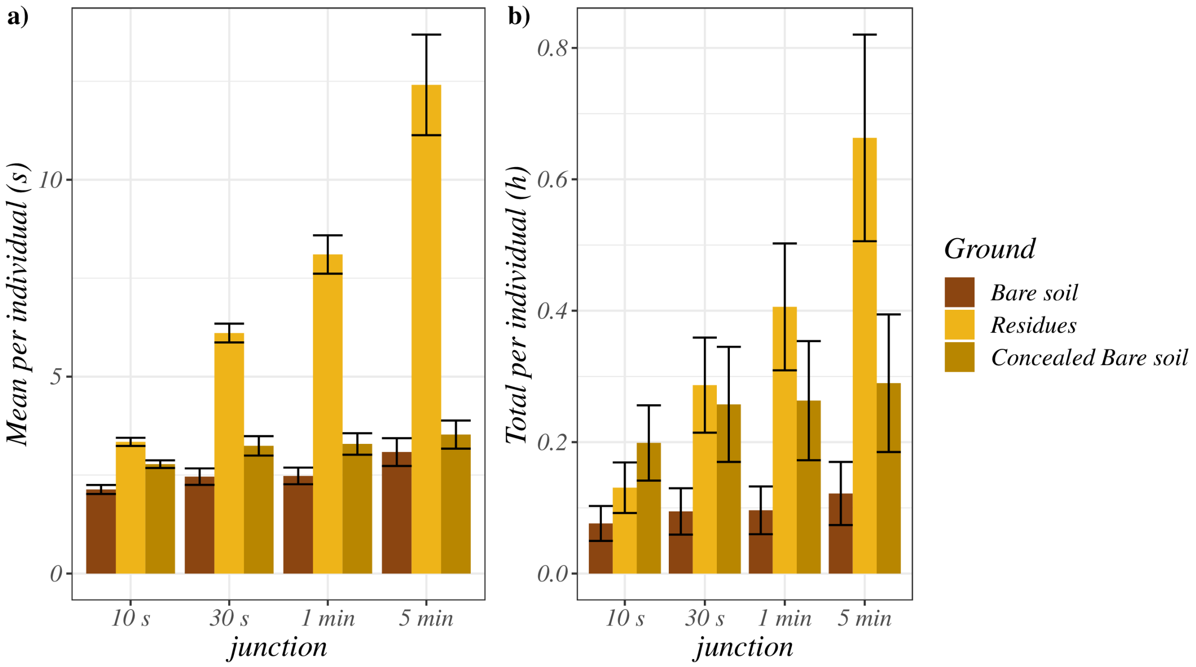

Fig J.2 : Statistics on time without observed movement per individual within trajectories (estimation of concealed duration) on a) mean duration (in seconds) or b) total duration (in hours). Inter-individual means are represented by coloured bars and black error bars represent standard errors.

Moreover, the pattern of response of concealed duration to time gaps for junction was very different for residues (**Fig. J.2**). On residues, both mean and total concealed duration per individual globally increased with longer junctions, with a concealed duration reaching a mean of 12 ± 5 s and a total of 39 ± 40 min (inter-individual mean ± standard deviation) (**Fig. J.2a** and **J.2b**). On concealed bare soil and bare soil, we noted a slight increase with longer junction for total and mean concealed duration but the magnitude stay very small, reaching for bare soil a mean of 3.1 ± 1.5 s and a total of 7.3 ± 11.9 min (inter-individual mean ± standard deviation) and for concealed bare soil, a mean of 3.5 ± 1.5 s and a total of 17.4 ± 25.9 min (inter-individual mean ± standard deviation) (**Fig. J.2a** and **J.2b**).

In conclusion, those results indicate that the long concealed periods we observed on residues cannot be explained only by the residues hiding part of the movement (as for concealed bare soil). This confirmed that the earwig's movements on residues have been different than those on bare soil.

References

Burr, M., 1939. Modern work on earwigs. Science Progress (1933-) 34, 20–30.

GIMP team, 1997. GNU Image Manipulation Program.

Joachim, C., Weisser, W.W., 2015. Does the aphid alarm pheromone (E)-beta-farnesene act as a kairomone under field conditions? J. Chem. Ecol. 41, 267–75.

Lamb, R.J., Wellington, W.G., 1974. Techniques for studying the behavior and ecology of the european earwig, Forficula auricularia (Dermaptera : Forficulidae). The Canadian Entomologist 106, 881–888. https://doi.org/10.4039/Ent106881-8

Turchin, P., 1998. Quantitative Analysis of Movement. Measuring and Modeling Population Redistribution in Animals and Plants. Sinauer Associates, Sunderland, Massachusetts.
