# Supplementary figures and images for: Assessing the effect of complex ground types on ground‐dwelling arthropod movements with video monitoring: Dealing with concealed movements under a layer of plant residues
Source: Ecol Evol. 2022 Jul 11;12(7):10.1002/ece3.9072. doi: 10.1002/ece3.9072 (PMC9271991; doi:10.1002/ece3.9072)

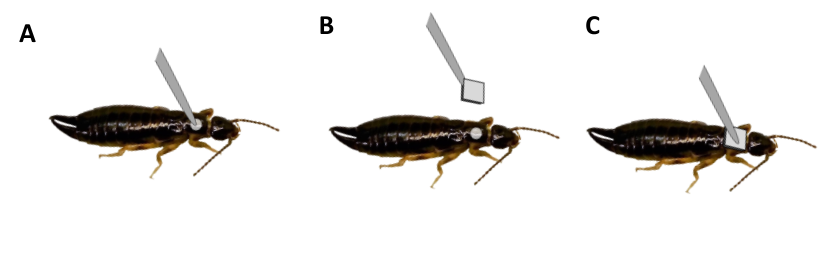

Supplement: Supplementary file 1 — Appendix S1. [file ECE3-12--s001.zip › ECE3_9072_Figure_C.1.png]

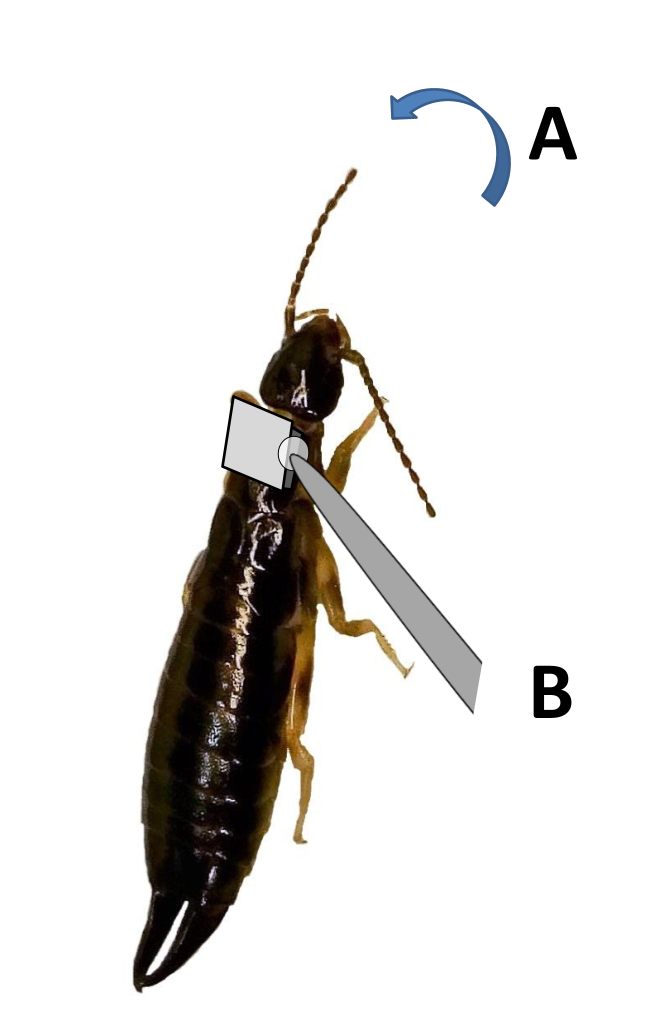

Supplement: Supplementary file 1 — Appendix S1. [file ECE3-12--s001.zip › ECE3_9072_Figure_C.2.png]

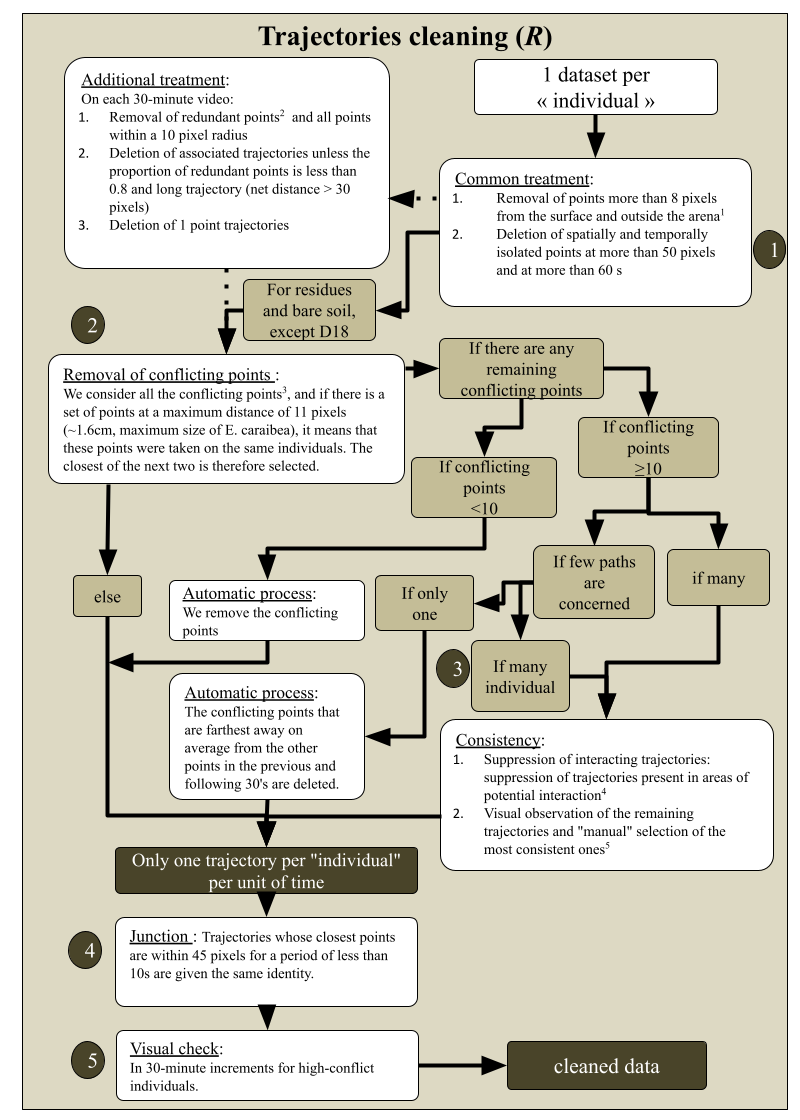

Supplement: Supplementary file 1 — Appendix S1. [file ECE3-12--s001.zip › ECE3_9072_Figure_E.1.png]

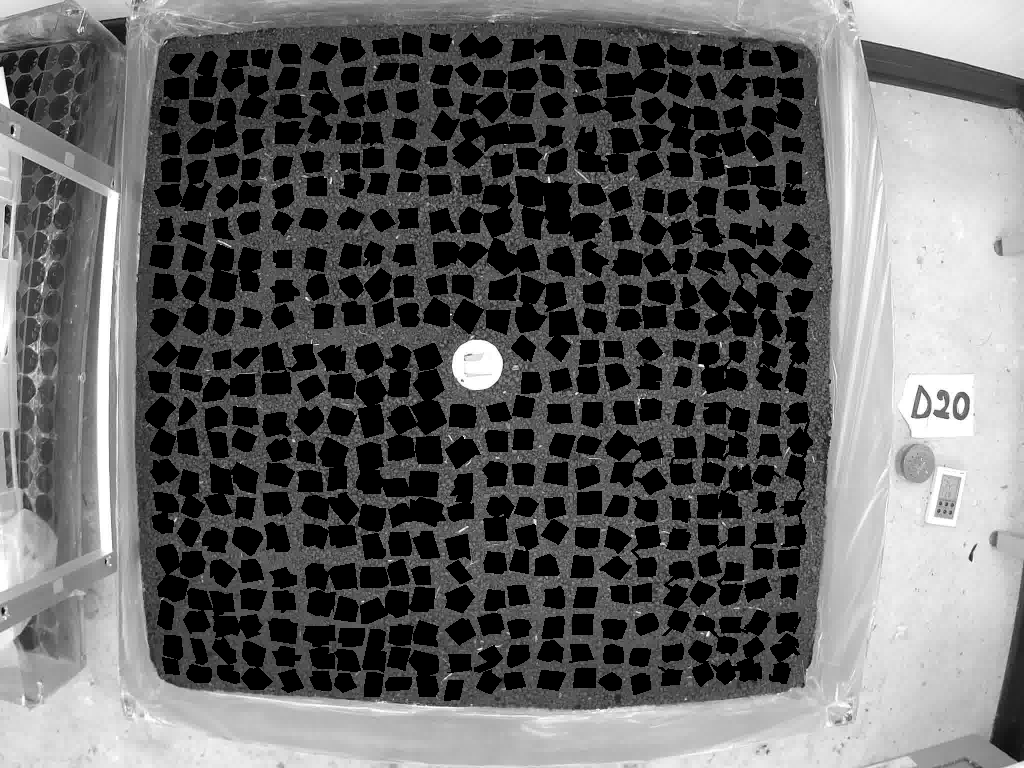

Supplement: Supplementary file 1 — Appendix S1. [file ECE3-12--s001.zip › ECE3_9072_Figure_J.1.png]

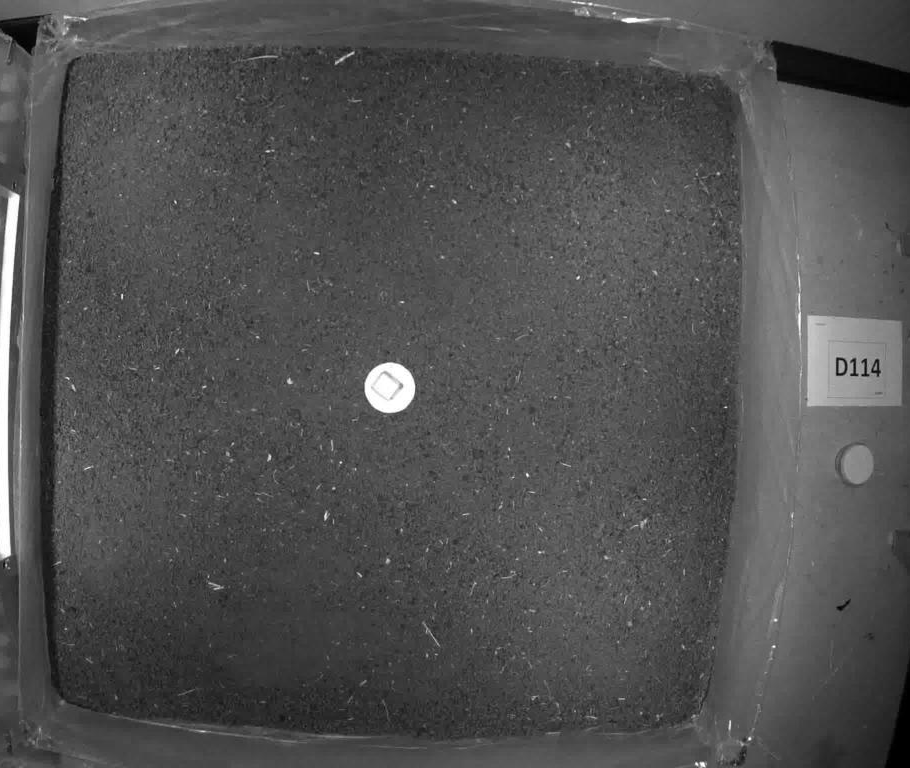

Supplement: Supplementary file 1 — Appendix S1. [file ECE3-12--s001.zip › ECE3_9072_Video_D.1.png]

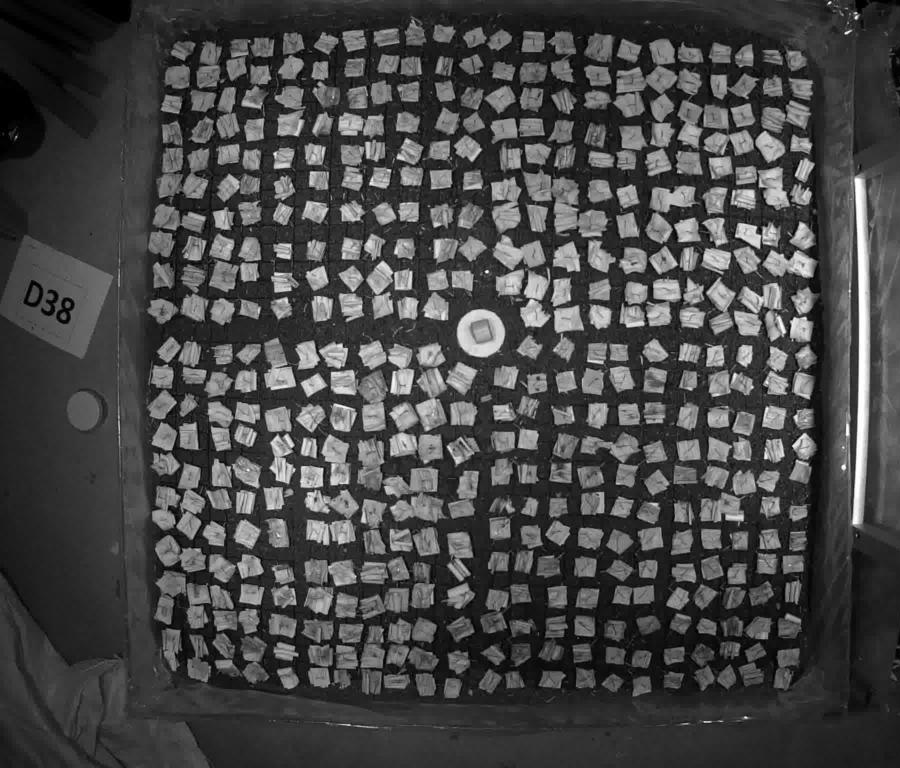

Supplement: Supplementary file 1 — Appendix S1. [file ECE3-12--s001.zip › ECE3_9072_Video_D.2.png]
